# Phosphoproteomic Profiling of Rat’s Dorsal Root Ganglia Reveals mTOR as a Potential Target in Bone Cancer Pain and Electro-Acupuncture’s Analgesia

**DOI:** 10.3389/fphar.2021.593043

**Published:** 2021-04-29

**Authors:** Wen Wang, You Zhou, Yangqian Cai, Sisi Wang, Fangbing Shao, Junying Du, Junfan Fang, Jinggen Liu, Xiaomei Shao, Boyi Liu, Jianqiao Fang, Yi Liang

**Affiliations:** ^1^ Key Laboratory of Acupuncture and Neurology of Zhejiang Province, Department of Neurobiology and Acupuncture Research, The Third Clinical Medical College, Zhejiang Chinese Medical University, Hangzhou, China; ^2^ Quzhou Municipal Hospital of Traditional Chinese Medicine, Quzhou, China

**Keywords:** proteomic profiling, protein microarray, mTOR, bone cancer pain, mechanism, electro-acupuncture

## Abstract

Bone cancer pain (BCP) is a clinical refractory mixed pain involving neuropathic and inflammatory pain, with the underlying mechanisms remaining largely unknown. Electro-acupuncture (EA) can partly alleviate BCP according to previous research. We aim to explore the proteins and major pathways involved in BCP and EA treatment through phosphoproteomic profiling. BCP rat model was built by tibial inoculation of MRMT-1 mammary gland carcinoma cells. Mechanical hyperalgesia determined by paw withdrawal thresholds (PWTs) and bone destruction manifested on the radiographs confirmed the success of modeling, which were attenuated by EA treatment. The differentially expressed phosphorylated proteins (DEPs) co-regulated by BCP modeling and EA treatment in rat dorsal root ganglions (DRGs) were analyzed through PEX100 Protein microarray. Kyoto Encyclopedia of Genes and Genomes (KEGG) analysis revealed that DEPs were significantly enriched in mammalian target of rapamycin (mTOR) signaling pathway. The phosphorylations of mTOR at Ser2448 and Thr2446 were increased in BCP and downregulated by EA. In addition, the phosphorylation of S6K and Akt, markers of the mTOR complex, were also increased in BCP and downregulated by EA. Inhibition of mTOR signaling alleviated the PWTs of BCP rats, while the mTOR agonist impaired the analgesic effect of EA. Thus, our study provided a landscape of protein phosphorylation changes in DRGs of EA-treated BCP rats and revealed that mTOR signaling can be potentially targeted to alleviate BCP by EA treatment.

## Introduction

Bone cancer pain (BCP) is caused by bone metastasis from tumor and has both pathological features of neuropathic pain and inflammatory pain, which remains clinically challenging. According to the latest estimates by the International Agency for Research on Cancer (IARC), 18.1 million new cancer cases and 9.6 million cancer deaths were reported worldwide in 2018 (Bray et al., 2018). With survival period prolonged, cancer pain has become one of the most important factors affecting the quality of life of cancer patients. The incidence of cancer pain in advanced or metastatic cancer patients who received anticancer treatment was reported as 66% (van den Beuken-van Everdingen et al., 2016; Bennett, 2017), of which bone metastases-induced cancer pain was the most common (Meuser et al., 2001). Breast cancer, prostate cancer, lung cancer, kidney cancer, thyroid cancer, and other cancers can all have bone metastasis and cause BCP, among which the incidence of bone metastasis of breast cancer is up to 70% (Coleman, 2001; Woodward et al., 2011).

At present, opioids are the main drugs for cancer pain treatment, and they are strongly recommended by the WHO for moderate and severe cancer pain control under the guidance of the three-step analgesic principle. However, opioid tolerance, opioid-induced hyperalgesia, and other side effects have always limited the widespread application of opioids in cancer pain. The adverse effects or the improper use of opioid drugs such as morphine was a matter of concern, which leads to inadequate control of cancer pain (Xia, 2017; Arthur and Reddy, 2019). According to incomplete statistics, the control rate of cancer pain in China is 43.8∼46.95%, which is lower than the global average (Li et al., 2017; Xia, 2017). Therefore, how to increase the efficiency of cancer pain management and explore a multiple-mode therapeutic regimen maintain to be a hot area in pain research.

Electro-acupuncture (EA) has been widely used in clinical analgesia and recommended as the main nondrug therapy for cancer pain treatment in the NCCN cancer treatment guidelines issued by the National Cancer Institute of the United States. At present, many clinical trials and experimental studies have confirmed the effectiveness of acupuncture in treating cancer pain (Paley et al., 2011; He et al., 2020b). Clinical trials have demonstrated that the efficacy of acupuncture in advanced cancer pain with unknown causes is superior to three-step drug therapy (Chen et al., 2008b; Paley et al., 2015). Animal experimental studies have also verified the validity of acupuncture in BCP (Ryu et al., 2014). The previous research of our group showed that EA with different frequencies or treatment intervals had good analgesic effects in treating BCP of rats (Du et al., 2015; Liang et al., 2018). However, the underlying mechanism remains unclear.

Dorsal root ganglion (DRG) neurons are primary sensory afferent neurons, which underwent maladaptive molecular changes under painful injuries, resulting in hypersensitivity and hyperexcitability of sensory neurons (peripheral sensitization) and are crucial in chronic pain mechanism (Berta et al., 2017). Studies have shown that the occurrence and maintenance of BCP are closely related to the abnormal or dysfunction of ion channels, receptors, and pain signaling pathways in DRG primary afferent neurons, such as P2X3 receptor, transient receptor potential vanilloid-1 (TRPV1) receptor, endothelin (ET) receptor, and prostaglandin (PG) receptor (Ahmad et al., 2018). Peripheral opioid receptors acting specifically on DRG neurons are sufficient to produce significant analgesic and anti-inflammatory effects without central nervous system-mediated side effects (Iwaszkiewicz et al., 2013). Therefore, research on peripheral mechanisms may be of great potential in BCP and helps to alleviate side effects of central nervous system. Besides, the phosphorylation of certain molecules in either central or peripheral nervous system contributes to hyperalgesia in BCP rats (Pareek et al., 2006; Hanamura et al., 2017). In this study, PEX100 protein microarray was used to map the phosphorylated protein profile of DRG in BCP rats, so as to explore potential target proteins for treating BCP.

## Materials and Methods

### Animals

Specific pathogen-free grade female Sprague–Dawley (SD) rats (160–180 g) were purchased from the laboratory of Animal Research Center of Zhejiang Chinese Medical University. The rats in this experiment were housed in a controlled environment (five rats per cage, temperature: 25 ± 2°C, humidity: 55 ± 5%, and light: 12 h light/dark cycle), with standard rodent food and distilled water *ad libitum*. The study protocol and experiments were approved by the Animal Ethics Committee of Zhejiang Chinese Medical University (NO. ZSLL-2014-195) and performed according to the National Institutes of Health Guide for the Care and Use of Laboratory Animals (NIH Publications No. 8023, revised 1978).

### Establishment of Bone Cancer Pain Rat Model

The BCP model was induced by implanting MRMT-1 mammary gland carcinoma cells into the left tibia according to the protocol of Medhurst et al. (Medhurst et al., 2002). The percentage of cell activity (more than 95%) was calculated by TC10 Automated Cell Counter (Bio-Rad Laboratories, Inc., Irvine, CA, United States), and the cell density was adjusted to 1 × 10^4^ cells/µl. First, the rats were anesthetized by isoflurane (2% output concentration) at a low oxygen flow rate of 500 ml/min. Then, they were placed in the supine position, with left hind limb shaved and sterilized. A 9-gauge needle was followed to puncture a hole in the upper tibia at an angle of 30∼45° from 5 mm below the tibial tubercle. Then, the carcinoma cells (3 × 10^4^ cells in 3 µl) were injected into the medullary cavity of the left tibia with a microsyringe (10 μl, Hamilton Co., Bonaduz, Switzerland). To prevent leakage, microsyringe was kept in position for another 1 min, and the hole was sealed immediately by bone wax. Finally, penicillin (20,000 units per rat) was injected intramuscularly after the surgery and was given once per day for 5 consecutive days to avoid infection. The rats in the control group received an equivalent amount of sterilized PBS instead of carcinoma cells.

### Experimental Design

**FIGURE 1:**
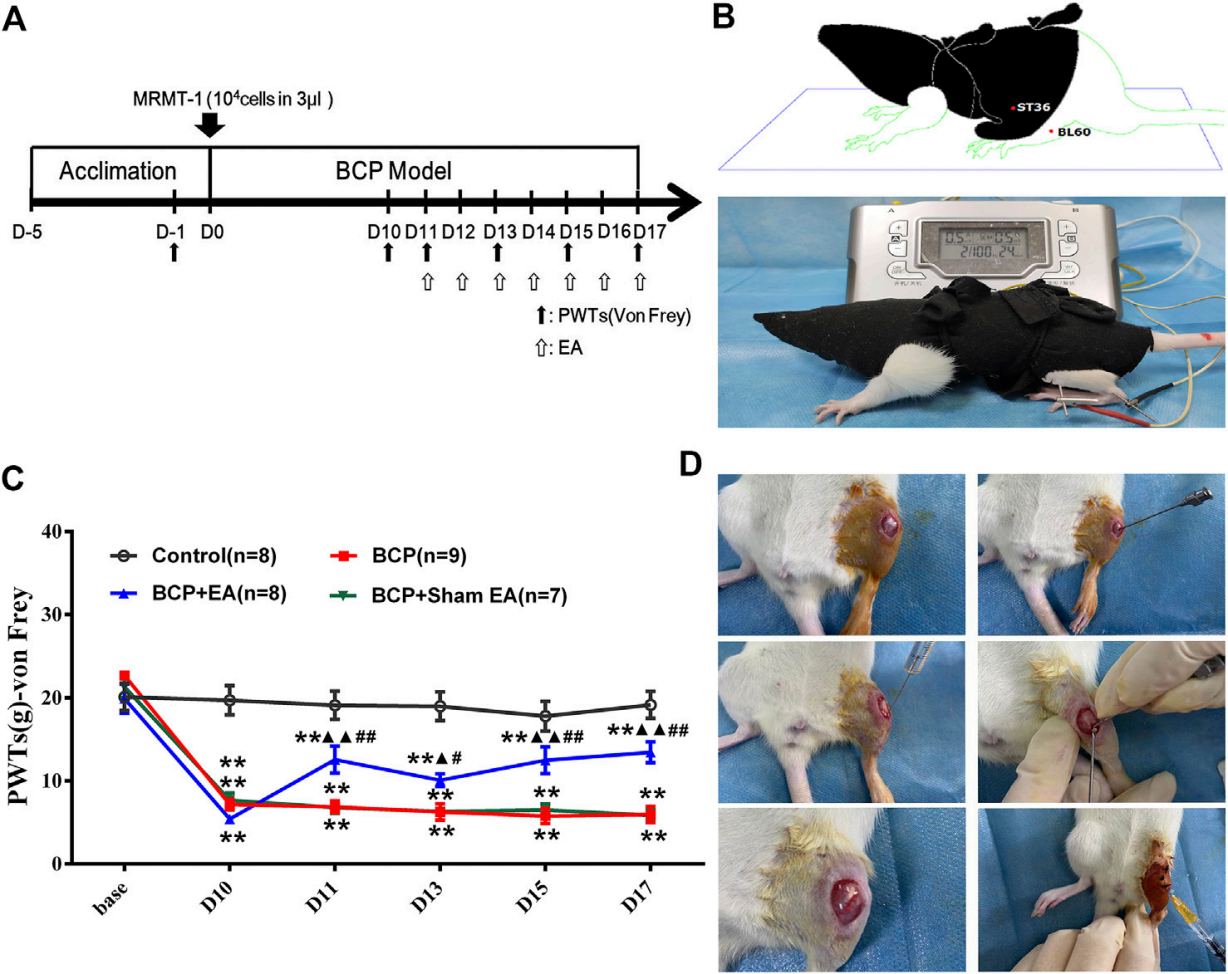
Establishment of the BCP rat model and time-course change of PWTs in rats. **(A)** Timeline of the animal experiment; **(B)** EA treatment on Zusanli (ST36) and Kunlun (BL60); **(C)** time-course change of PWTs. Compared with the control group, PWTs of the BCP, BCP + EA, and BCP + Sham EA groups dropped drastically on postoperative day 10 and kept decreasing until day 17, indicating successful modeling. The PWTs of the BCP + EA group increased after intervention of EA on postoperative days 11, 13, 15, and 17. **(D)** Establishment of the BCP model. PWTs: pain withdrawal thresholds; EA: electro-acupuncture; BCP: bone cancer pain; base: baseline. **p < 0.01, compared with the control group; ▲ *p* < 0.05, ▲▲ *p* < 0.01, compared with the BCP group; #*p* < 0.05, ##*p* < 0.01, compared with the BCP + Sham EA group.

The experiment consisted of three series. In series 1, we investigated the time-course changes of PWTs value after the establishment of the rat BCP model or treatment of EA or sham EA. After 5 days of adaptive feeding, the rats for establishment of BCP model were randomly allocated as follows: 1) the control group, which received vehicle (PBS) inoculation without carcinoma cells; 2) the BCP group, which received intramedullary inoculation of MRMT-1 mammary gland carcinoma cells (3 × 10^4^ cells in 3 µl) in the upper left tibia; 3) the BCP + EA group, which received the same inoculation as the BCP group and EA treatment (dilatational wave, 2/100 Hz, 7 times in total); and 4) the BCP + Sham EA group, which also received inoculation of carcinoma cells (same as the BCP group) and sham EA treatment (acupuncture subcutaneously, with no electrode connected, seven times in total). Pain behavioral test and EA treatment were conducted according to the schedule (Figure 1A), i.e., PWT test on days −1, 10, 11, 13, 15, and 17, and EA treatment from postoperative days 11–17. The PWT test was conducted immediately after EA treatment in 1 h. All rats were euthanized at the end of the experiment, and the relevant tissues were excised for the following study. Among these, a part of rats in the control, BCP, and BCP + EA groups was randomly selected to do the PEX100 protein microarray assay.

In series 2, the effect of mTOR-specific inhibitor rapamycin on the PWTs of BCP rats was observed. In brief, 20 rats were randomly divided into two groups (n = 10 per group), namely, BCP rats with an i.t. injection of 10 μg/10 μl rapamycin 45 min before behavior test on postoperative days 11–17 (BCP + rapamycin group), and BCP rats with an i.t. injection of 10 μl sterilized saline instead of rapamycin (BCP + vehicle group). Pain behavioral test was conducted on days −6, −1, 10, 11, 13, 15, and 17.

In series 3, the involvement of mTOR-specific agonist L-leucine in EA-mediated pain regulation was explored. In brief, 20 rats were randomly divided into two groups (n = 10 per group), namely, BCP + EA + L-leucine rats with an i.t. injection of 100 μg/10 μl L-leucine 45 min before behavior test on postoperative days 11–17 (BCP + EA + L-leucine group), and BCP + EA rats with an i.t. injection of 10 μl sterilized saline (BCP + vehicle group). Pain behavioral tests were conducted on days −6, −1, 10, 11, 13, 15, and 17, and EA treatment from postoperative days 11–17. The PWT test was conducted immediately after EA treatment in 1 h.

### Drug Preparation and Intrathecal Administration

Rapamycin (Sigma-Aldrich, Saint Louis, MO, United States), a selective mTOR inhibitor, was dissolved in 20% dimethyl sulfoxide (DMSO) to prepare stock solution (stored at −20°C). L-leucine (APExBIO, Houston, TX, United States), a selective mTOR agonist, was dissolved in H_2_O to prepare stock solution (stored at −20°C). They were diluted to the requested concentrations by sterilized 0.9% saline before treatment and were administered intrathecally from days 11 to 17 after tumor cell inoculation for 7 consecutive days. The dose of rapamycin was 10 μg/10 μl and L-leucine was 100 μg/10 μl. In previous research, the different intrathecal doses (0.1, 1, 10 μg or 1, 5, 10 μg) of rapamycin were used, and it dose-dependently attenuated the development and maintenance of BCP (Shih et al., 2012; Jiang et al., 2016). Therefore, 10 μg/10 μl for rapamycin was the most appropriate dose. And for L-leucine, 100 μg was the maximal and safe intrathecal dose; it was used to observe the inversion of the effects of other drugs (Cheng et al., 2000). In our study, L-leucine was adopted to observe its inversion of EA’s analgesia. Therefore, 100 μg/10 μl for L-leucine was enough and safe dosage for intrathecal utilization. The vehicle treatment was sterilized 0.9% saline. All the drugs were delivered in a volume of 10 μl solution, followed by an addiction of 25 μl saline to flush the catheter.

Intrathecal catheterization was carried out according to the method of Yu-Qiu Zhang et al. and Luc Jasmin et al. (Jasmin and Ohara, 2001; Zhang et al., 2002). In brief, animals were anesthetized by isoflurane (2% output concentration) at a low oxygen flow rate of 500 ml/min. The PE-10 catheter was inserted 2 cm cephalad into the lumbar subarachnoid space at the level of L4–L5 intervertebral space. The spinal cord of rats generally terminated at the level of L3–4 intervertebral space (Padmanabhan and Singh, 1979), and the tip of the catheter was located at the cauda equina region. The applied chemicals were limited at the L4–L5 DRG levels by using a small volume (10 μl) of the injectant and limiting the rate of injection (3.3 μl/min) (Chen et al., 2008a). The catheter was tunneled subcutaneously and externalized through the skin in the neck region. Penicillin (20,000 units per rat) was injected intramuscularly (once per day) after the catheterization for 5 consecutive days to avoid infection. One day after the catheterization, 20 µl of 2% lidocaine was injected intrathecally to the rats without impaired movement. The successful sign for catheterization was lower limb paralysis within 30 s after injection, otherwise the rats were eliminated. PWT tests were utilized on days −6 and −1 to make sure that the intrathecal catheterization had no influence on baseline of mechanical pain.

### Electro-Acupuncture Treatments

As for EA treatment, rats in the BCP + EA group were bounded by a special cotton retainer designed by our laboratory (Patent No. ZL 2014 2 0473579.9, State Intellectual Property Office of the People’s Republic of China). The bilateral acupoints of ST36 (Zusanli, 5 mm lateral to the anterior tubercle of the tibia bone) and BL60 (Kunlun, localized at the ankle joint level, between the tip of the lateral malleolus and the achilles tendon) were selected. Then the sterilized disposable acupuncture needles (0.25 mm in diameter, 13 mm in length, Suzhou Medical Appliance Factory, Suzhou, China) of stainless steel were inserted bilaterally to ST36 and BL60 at the depth of 5 mm. The two homo-lateral needles were connected to two electrodes of the same output terminal on the HANS Acupuncture Point Nerve Stimulator (Hans-100, Huawei Co., Ltd., Beijing, China). The EA parameters were set as follows: dilatational wave (pulse width: 0.6 ms at 2 Hz, 0.2 ms at 100 Hz, automatically shifting between 2 and 100 Hz stimulation every 3 s); intensities of 0.5-1-1.5 mA (10 min for each, 30 min in total). The EA stimulation was given every day for 30 min from postinoculation days 11 to 17, seven times in total. As for sham EA treatment, the rats were immobilized as well and the needles were inserted subcutaneously with no electrodes connected. The frequency and duration of treatment were the same. The EA parameter was selected according to the results of our previous research in which different frequencies (2, 100, and 2/100 Hz) combined with different intervals (once a day or once every other day) of EA were utilized in relieving BCP. However, the analgesic effects of EA were not related to frequency and interval (Du et al., 2015). As BCP involves neuropathic and inflammatory pain, 2/100 Hz was used because 2 Hz has advantage on neuropathic pain and 100 Hz on inflammatory pain (Jiang et al., 2001; Du et al., 2020). Besides that, our group had identified the efficacy of 2/100 Hz in relieving BCP previously (Du et al., 2015; Liang et al., 2018), and the dilatational wave was recommended to avoid acupuncture tolerance.

### Measurement of Pain Behavior

PWTs were carried out by using von Frey behavioral test, according to the up–down method described by Chaplan et al. and our previous research (Chaplan et al., 1994; Xiang et al., 2019). Rats were acclimatized to the testing environment 30 min daily for continuous 3 days before baseline test. Before each test, rats were placed into the transparent cage for at least 30 min to acclimate. The von Frey filaments (Stoelting Co., Thermo, Gilroy, CA, United States) were applied through consecutive ascending order (4, 6, 8, 15, and 26 g) and punctured perpendicularly to the center of the left hind paw, sustaining for at least 5 s. An abrupt withdrawal or paw flinching was considered as a positive response “X.” Then a weaker stimulus was replaced. In the case of no reaction, “O” was recorded, followed by a stronger stimulus. Each stimulus was spaced more than 2 min apart. When the first “X” appeared, a series of another four stimuli were applied until done. The formula was the same as previously described. The PWTs of baseline and postinoculation days 10, 11, 13, 15, and 17 were measured. For experiment series 1 and 3, the PWTs of rats were conducted within 1 h after the completion of EA or Sham EA treatment on postinoculation days 11, 13, 15, and 17. The pain measuring time was fixed at 9:00–14:00, and the ambient temperature was 23 ± 2°C.

### X-Ray Detection

Rats were anesthetized by using sodium pentobarbital (1.5 ml/kg, i.p.) on postoperative day 16 and exposed to PLX7000B HF mobile C-arm X-ray equipment for 5 s at 50 kV, 0.6 mA (Perlove Medical Equipment Co., Ltd., Nanjing). The rats were put in the supine position on the motor frame and X-ray imaging was performed on the ipsilateral side of the rat hind limbs to detect any bone changes in the proximal tibia based on blind analysis.

### PEX100 Protein Microarray Assay

Phosphoprotein profiling by the Phospho Explorer Antibody Array PEX100, which was designed and manufactured by Full Moon Biosystems, Inc. (Sunnyvale, CA), contains 1318 antibodies. Each of the antibodies has two replicates that are printed on coated glass microscope slide, along with multiple positive and negative controls. The antibody array experiment was performed by Wayen Biotechnology (Shanghai, China) according to the established protocol.

The cancer cells were inoculated in the left tibia, and the PWT measurement was conducted on the left hind paw. The lower hind limb of rats was innervated by sciatic nerve, which consisted of L4, L5, and L6, whereas L3 and L4 constituted the femoral nerve and L2–L4 mainly innervated the quadriceps femoris (Wessels et al., 1990). DRG neurons are primary sensory afferent neurons, playing an important role in the development and maintenance of BCP (Ahmad et al., 2018). Peripheral opioid receptors acting specifically on DRG neurons exerted analgesic and anti-inflammatory effects without central nervous system-mediated side effects (Iwaszkiewicz et al., 2013). In addition, our research group had found that various pain conditions including neuropathic pain and inflammatory pain were related to molecular mechanisms in L4–L6 DRGs (Li et al., 2019; Xiang et al., 2019). Therefore, on postoperative day 17 after PWT measurement, the left L4–L6 DRGs of rats were collected after transcardial perfusion of sterilized saline (4°C). Then, the left L4–L6 DRGs of several rats from the control, BCP, and BCP + EA groups were used to obtain tissue lysates according to Full Moon’s standard practice process, and the tissue lysates were biotinylated with the Antibody Array Assay Kit (Full Moon Biosystems, Inc.). The antibody microarray slides were first blocked in a blocking solution (Full Moon Biosystems, Inc.) for 30 min at room temperature, rinsed with Milli-Q grade water for 3–5 min, and dried with compressed nitrogen. The slides were then incubated with the biotin-labeled cell lysates (∼100 μg protein) in coupling solution (Full Moon Biosystems, Inc.) at room temperature for 2 h. The array slides were washed 4–5 times with 1X wash solution (Full Moon Biosystems, Inc.) and rinsed extensively with Milli-Q grade water before detection of bound biotinylated proteins by using Cy3-conjugated streptavidin. The slides were scanned on a GenePix 4000 scanner and the images were analyzed by using GenePix Pro 6.0 (Molecular Devices, Sunnyvale, CA). The fluorescence signal (I) of each antibody was obtained from the fluorescence intensity of this antibody spot. A ratio computation was used to measure the extent of protein phosphorylation. The phosphorylation level of the protein at that phosphorylated site was obtained by specific phosphorylated antibody signal value divided by nonphosphorylated antibody signal value, namely, $\text{ phosphorylation ratio}=\frac{\text{phospho value}}{\text{unphospho value}}$. The intergroup modulation of phosphorylation was calculated by $\text{phosphorylation ratio}=\frac{\text{phospho ratioExp}\text{.}}{\text{ phospho ratioCon}\text{. }}$. The datasets presented in this study can be found in online repositories. The names of the repository and accession number can be found as follows [protein microarray database and PMDE256].

### Bioinformatics Analysis

In all, 584 couples of phosphorylated proteins and nonphosphorylated proteins were detected in Phospho Explorer Antibody Array PEX100. In general, the number of DEPs between groups was limited to 10–30% of the total detected protein number. Therefore, the fold change threshold was set as 1.6 in our research. According to these criteria, the numbers of DEPs were 119 between the BCP and control groups, 176 between the BCP + EA and BCP groups, and 58 between the BCP + EA and control groups. Upregulation or downregulation of 15, 20, and 50% could also be chosen according to the published chip literature (Kang et al., 2010). The KEGG pathway and GO enrichment were performed by using DAVID analysis (https://david.ncifcrf.gov/home.jsp). The modified Fisher’s exact *p* value here was for gene-enrichment analysis, and *p* < 0.05 was considered as strongly enriched in the annotation categories.

### Western Blot

The phosphorylation of mTOR, S6K, and Akt was measured by Western blot. Rats were rapidly sacrificed on postoperational day 17 by heart-perfusion of saline. L4–L6 DRGs were quickly harvested and lyzed by RIPA buffer; the lysates were centrifuged at 14,000 g for 5 min at 4°C. Supernatants were collected, and the total protein concentration was titrated by using the BCA protein assay regent kit. Equivalent amounts of protein (20 μg) were fractionated on 8% polyacrylamide gels. Proteins were transferred to the polyvinylidene fluoride (PVDF) membranes (Merck KGaA, Darmstadt, Germany) at 0.4 A for 2 h. Membranes were blocked with 5% skim milk in Tris-buffered saline Tween (TBST) for 1 h at room temperature, then incubated overnight at 4°C with corresponding primary antibody (anti-mTOR at 1:1000, Abcam; anti-p-mTOR Ser2448 at 1:1000, CST; anti-p-mTOR Ser2481 at 1:1000, Abcam; anti-p-mTOR Thr2446 at 1:1000, OriGene; anti-GAPDH at 1:2000, Jackson; anti-Akt at 1:1000, CST; anti-p-Akt ser473 at 1:2000, Abcam; anti-S6K at 1:5000, Abcam; and anti-p-S6K Thr389 at 1:1000, CST) diluted in TBST. Following three washes with TBST, membranes were incubated with horseradish peroxidase (HRP)–conjugated secondary antibodies (1:5000, CST) in TBST for 2 h at room temperature, then with another three washes of TBST. Western blot strips were visualized by using ECL through an Image Quant LAS 4000 gel imaging system. The gray values of the strips were quantified by Image Quant TL software. The GAPDH was utilized as loading control.

### Statistical Analysis

All data are expressed as means ± standard error of the mean (SEM). The statistical analyses were conducted by using SPSS 17.0. The PWTs were analyzed by two-way analysis of covariance with repeated measures, followed by Bonferroni’s *post hoc* test to compare the difference between groups or different time points. The other data were compared by a one-way analysis of variance followed by Bonferroni’s *post hoc* analysis. *p* < 0.05 was considered as statistically significant.

## Results

### Electro-Acupuncture Relieves Mechanical Pain Behavior of Bone Cancer Pain Rats

After 5 days of acclimation, the rats were inoculated with MRMT-1 mammary gland carcinoma cells or sterilized PBS. After the establishment of the BCP model, EA or sham EA was adopted from postoperative days 11 to 17 (Figures 1A,B). The PWTs of rats were obviously decreased 10 days after BCP operation compared with the control group and kept steadily dropping till postoperative day of 17 (*p* <0.01), which was consistent with previous research (Figure 1C). EA treatment increased the PWTs on days 11, 13, 15, and 17 after carcinoma cell inoculation (*p* < 0.01 or *p* < 0.05). However, Sham EA treatment showed no obvious modulation on PWTs compared with the BCP group (Figure 1C). The experimental procedure of BCP modeling is shown in Figure 1D.

### Obvious Bone Destruction Was Observed in the Tibial Bone After Inoculation of Carcinoma Cells From Radiographs

**FIGURE 2:**
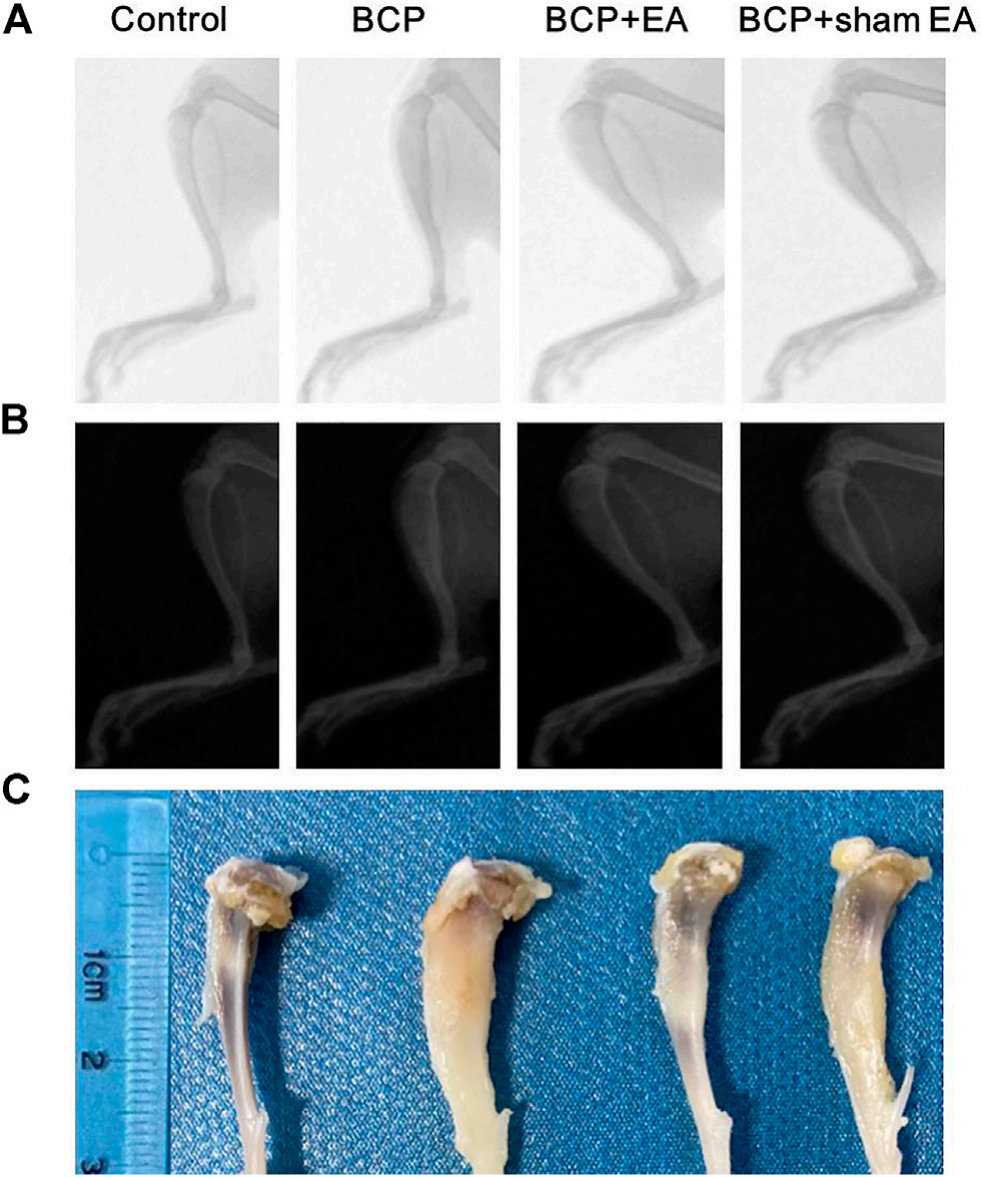
Radiographs of the tibial bone inoculated with PBS (control) or MRMT-1 mammary gland carcinoma cells (BCP). **(A)** X-ray radiographs of the ipsilateral tibial bone on the 16th day after carcinoma cell inoculation; **(B)** the invert X-ray radiographs of the ipsilateral tibial bone on postoperative day 16; **(C)** gross anatomy of the ipsilateral rat tibia. The scale ruler was shown on the left of the tibia bones.

Radiological examination was used to observe bone destruction in the rat model of BCP. As shown by X-ray images, no obvious alternation of tibia structure was found at 16th day after inoculation of sterilized PBS (control rats). However, 16 days after inoculation of MRMT-1 mammary gland carcinoma cells, obvious bone destructions were observed at about one-third of the left tibia in BCP, BCP + EA, and BCP + sham EA rats, manifested by bone trabecula defects and loss of normal bone structure (Figures 2A,B). Compared with BCP and BCP + sham EA rats, the bone destruction in EA-mediated rats was alleviated to some extent from radiological images and anatomical visual observation (Figures 2A–C).

### Phosphoproteomic Profiles of Rat Dorsal Root Ganglions Demonstrated Significant Changes in Protein Phosphorylation After Bone Cancer Pain Modeling or Electro-Acupuncture Treatment

**FIGURE 3:**
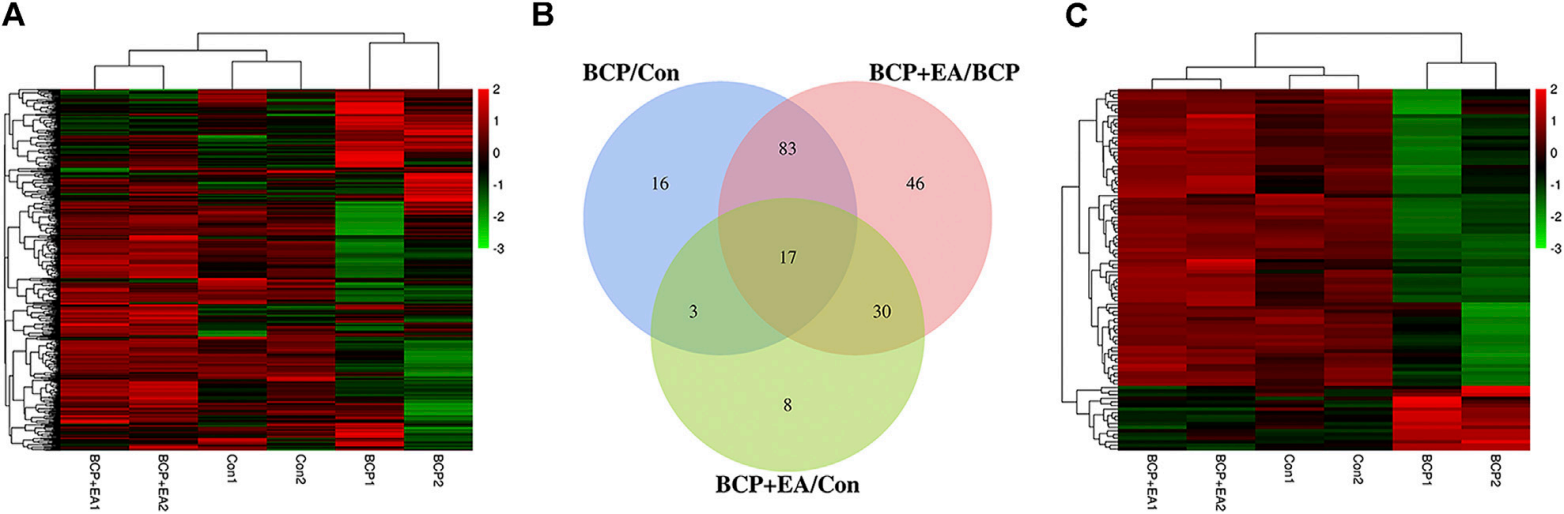
Phosphoproteomic profile of rat DRGs detected by the phosphor-explorer array PEX100 assay. (**A)** Heat map illustration of hierarchical clustering of overall phosphoproteomic profile of DRGs in rats. **(B)** Venn diagram showing DEPs in DRGs between different groups. BCP/Con stands for DEPs between the BCP and control groups, and the others are in a similar fashion. **(C)** Heat map illustration of hierarchical clustering of DEPs between groups. Con: Control; DRGs: dorsal root ganglions.

Phospho Explorer Antibody Array PEX100 was used to explore phosphoproteomic profiles of ipsilateral L4–6 DRGs 17 days after BCP modeling or EA treatment. In total, 584 pairs of coupled antibodies were detected, with each phosphorylation site detected by phosphorylated and nonphosphorylated states. Then the phosphorylation level of the protein was calculated by phosphorylation ratio. Overall, the hierarchical clustering analyses were utilized to get an overview of protein phosphorylation profiles of control, BCP, and BCP + EA rats, showing that samples within each group were clustered in their separate groups (Figure 3A). The clear segregation and clustering of the data indicated that distinct protein phosphorylation existed between groups but not within groups. Then the folder change threshold of 1.6 was selected to filter DEPs. The numbers of DEPs were 119 between the BCP and control groups, 176 between the BCP + EA and BCP groups, and 58 the between the BCP + EA and control groups, and the DEP intersection of BCP/Con and BCP + EA/BCP was 100 (Figure 3B). The selected DEPs were further illustrated in a heat map with clustering analysis, which indicated a high level of consistency in either the control group or BCP group or BCP + EA group samples (Figure 3C). The above data demonstrated that BCP modeling or EA treatment would result in significant phosphorylation changes in the ipsilateral DRGs of rats. In addition, the fold change and *p* value of the overall phosphorylated proteins and DEPs are respectively shown in Supplementary Tables S1 and S2. The differential phosphorylation of proteins after BCP modeling or EA treatment based on both fold change and *p* value is shown in Supplementary Figure S1. However, the fold change was mainly concerned with PEX100 Protein microarray assay (Chen et al., 2020; He et al., 2020a).

### Gene Ontology (GO) Analysis of Differentially Expressed Phosphorylated Proteins Co-Regulated by Both Bone Cancer Pain Modeling and Electro-Acupuncture Treatment in Rat Dorsal Root Ganglions

**FIGURE 4:**
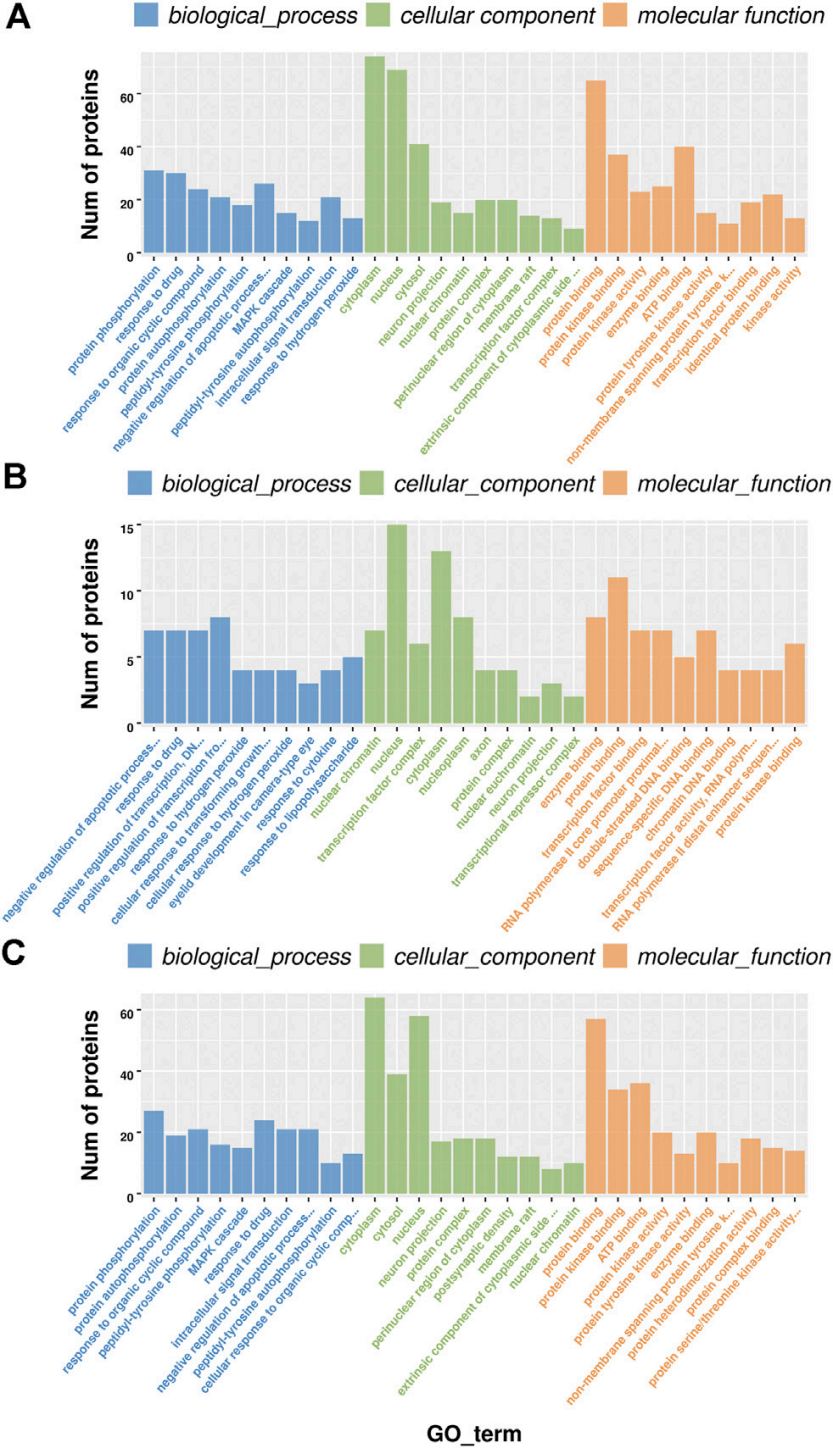
GO analysis of DEPs co-regulated by both BCP modeling and EA treatment in rat DRGs. **(A)** The top 10 significant biological processes, molecular functions, and cellular components of DEPs shared by both the BCP and BCP + EA groups; **(B)** the top 10 significant biological processes, molecular functions, and cellular components of DEPs upregulated in the BCP group but further downregulated by EA intervention in the BCP + EA group; **(C)** the top 10 significant biological processes, molecular functions, and cellular components of DEPs downregulated in the BCP group but further upregulated by EA intervention in the BCP + EA group; the significant *p*-value was 0.05. GO: gene ontology; DEPs: differentially expressed phosphorylated proteins.

To further explore the underlying molecular mechanism of BCP modeling and EA treatment, the GO analysis was carried out based on DEPs between groups. The selected DEPs were those co-regulated by both BCP modeling and EA treatment. It happened to be either upregulated in the BCP group but further downregulated by EA intervention in the BCP + EA group or inverse. The GO analysis mainly includes biological process, cellular component, and molecular function. The most significant data were included to do histogram analysis. In general, the most enriched biological process was protein phosphorylation, response to drug, negative regulation of apoptotic process, etc.; the most abundant cellular component was cytoplasm, nucleus, cytosol, etc.; the most concentrated molecular function was protein binding, ATP binding, protein kinase binding, etc. (Figure 4A). For those DEPs upregulated in the BCP group and downregulated by EA treatment in the BCP + EA group, the most enriched biological process was positive regulation of transcription from RNA polymerase II promoter, negative regulation of apoptotic process, response to drug, etc.; the most abundant cellular component was nucleus, cytoplasm, nucleoplasm, etc.; the most concentrated molecular function was protein binding, enzyme binding, transcription factor binding, etc. (Figure 4B). For those DEPs downregulated in the BCP group and further upregulated by EA in the BCP + EA group, the most enriched biological process was protein phosphorylation, response to drug, response to organic cyclic compound, etc.; the most abundant cellular component was cytoplasm, nucleus, cytosol, etc.; the most concentrated molecular function was protein binding, ATP binding, protein kinase binding, etc. (Figure 4C).

### Kyoto Encyclopedia of Genes and Genomes Pathway Analysis of Differentially Expressed Phosphorylated Proteins in Rat Dorsal Root Ganglions Revealed That Mammalian Target of Rapamycin Pathway and Mammalian Target of Rapamycin Phosphorylation Were Involved in Electro-Acupuncture-Mediated Analgesic Effects on Bone Cancer Pain

**FIGURE 5:**
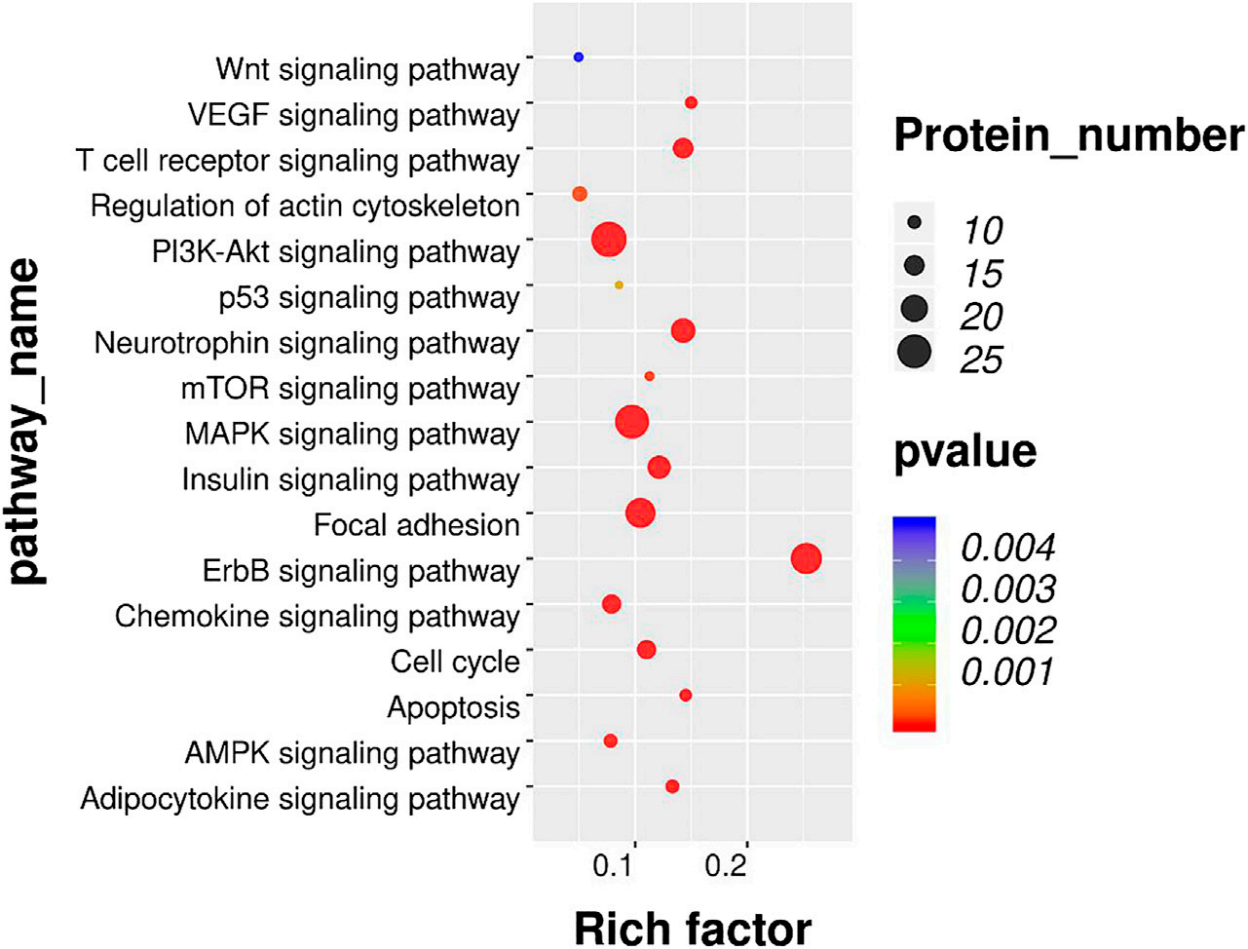
KEGG pathway analysis of DEPs co-regulated by both BCP modeling and EA treatment in rat DRGs. Bubble plots showing the significant KEGG pathways for DEPs with opposite modulating trend in the BCP and BCP + EA groups. The color of each bubble represents significance (*p*-value), and the volume of each bubble represents protein number. KEGG: Kyoto Encyclopedia of Genes and Genomes; DEPs: differentially expressed phosphorylated proteins.

To further investigate the enriched pathway of DEPs, the KEGG pathway analysis was adopted. As shown in Figure 5, the DEPs with opposite modulating trend in the BCP and BCP + EA groups (either upregulated in the BCP group and further downregulated by EA treatment in the BCP + EA group or downregulated in the BCP group and further upregulated by EA treatment in the BCP + EA group) were mainly involved in 17 KEGG pathways of PEX100 protein-chip. The most significant pathways as for *p*-value were ErbB signaling pathway, MAPK signaling pathway, focal adhesion, PI3K-Akt signaling pathway, etc.; the most protein enriched pathways were PI3K-Akt signaling pathway, MAPK signaling pathway, ErbB signaling pathway, etc. From all the above, ErbB signaling pathway, PI3K-Akt signaling pathway, and MAPK signaling pathway were closely related with BCP or cancer and have been studied a lot. mTOR signaling pathway was the downstream of both ErbB and PI3K-Akt signaling pathway and the mTOR phosphorylation at Ser2481, Ser2448, and Thr2446 showed obvious opposite trend in the BCP group and the BCP + EA group (Supplementary Figure S2).

### The Phosphorylation of Mammalian Target of Rapamycin and Mammalian Target of Rapamycin Complex Markers Was Involved in Alleviating Bone Cancer Pain by Electro-Acupuncture Treatment

**FIGURE 6:**
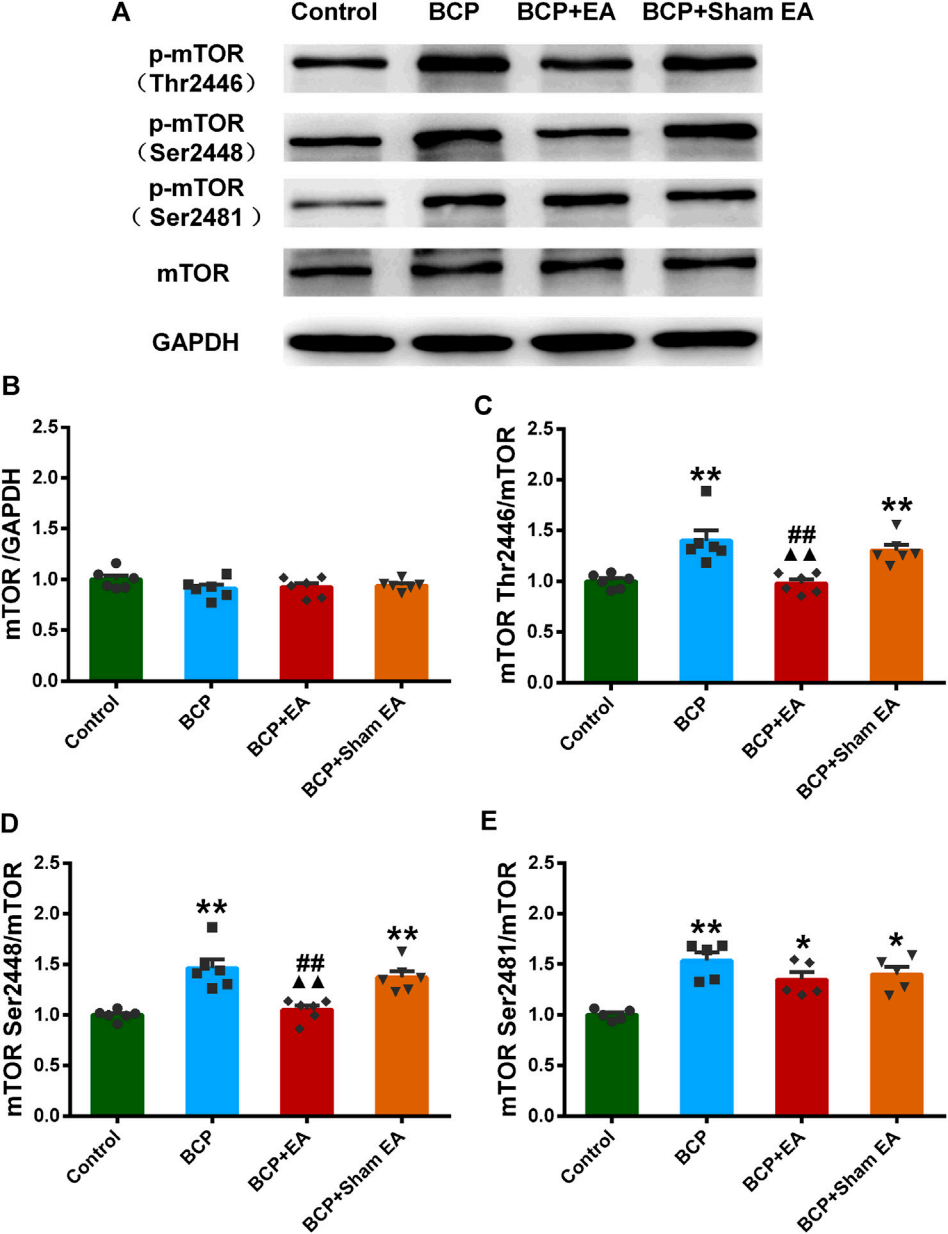
Regulation effects of EA on the phosphorylation of mTOR (Thr2446, Ser2448, and Ser2481). **(A)** The representative WB band; **(B)** the expression of mTOR total protein, N = 6 rats/group; **(C)** the expression of p-mTOR Thr2446, N = 6 rats/group; **(D)** the expression of p-mTOR Ser2448, N = 6 rats/group; **(E)** the expression of p-mTOR Ser2481, N = 5 rats/group. *p < 0.05, **p < 0.01, compared with the control group; ▲▲ *p* < 0.01, compared with the BCP group; ##*p* < 0.01, compared with the BCP + sham EA group.

**FIGURE 7:**
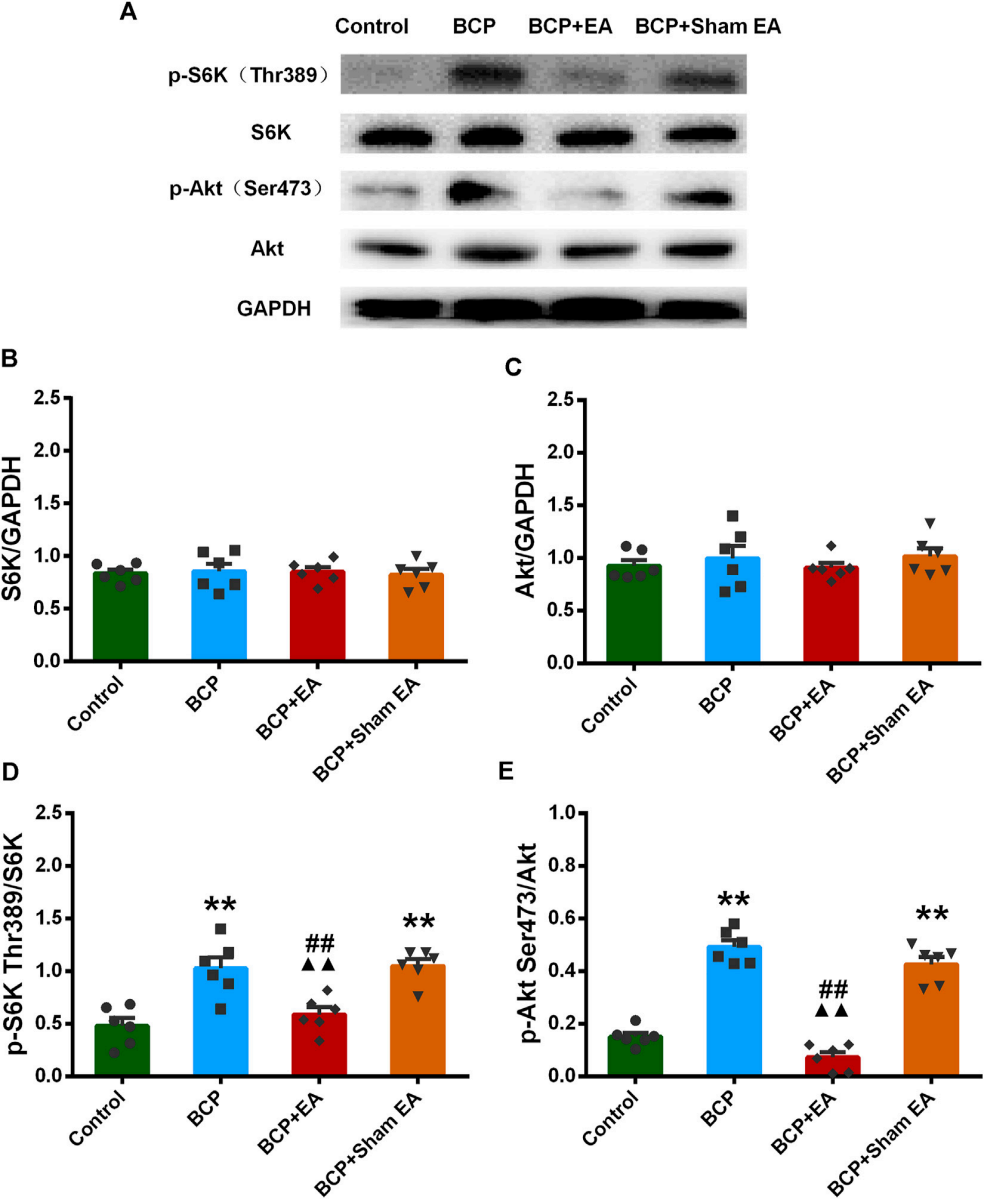
Regulation effects of EA on the phosphorylation of S6K and Akt. **(A)** The representative WB band; **(B)** the expression of S6K total protein, N = 6 rats/group; **(C)** the expression of Akt total protein, N = 6 rats/group; **(D)** the expression of p-S6K Thr389, N = 6 rats/group; **(E)** the expression of p-Akt Ser473, N = 6 rats/group. *p < 0.05, **p < 0.01, compared with the control group; ▲▲ *p* < 0.01, compared with the BCP group; ##*p* < 0.01, compared with the BCP + sham EA group.

We proceeded to verify the phosphoproteomic profiling data by Western blot. The phosphorylation site of mTOR at Ser2448, Ser2481, and Thr2446 was selected and further verified (Figure 6A). In phosphoproteomic profiling data, the phosphorylation of mTOR Ser2448 was increased in BCP rats (*p* < 0.05) and showed a downward trend after EA treatment. The phosphorylation of mTOR Thr2446 showed an upward trend after BCP modeling and a downward trend after EA treatment. However, the phosphorylation trend of mTOR (Ser2481) was totally the opposite, which was decreased in BCP rats (*p* < 0.05) and upregulated by EA (*p* < 0.05) (Supplementary Figure S2). WB results showed that the mTOR total protein remained unchanged between different groups; thus, neither BCP modeling nor EA treatment regulated the mTOR total protein expression (Figure 6B). The phosphorylated mTOR at Ser2448 and Thr2446 was upregulated in the BCP and BCP + Sham EA groups compared with the control group, whereas downregulated by EA treatment in the BCP + EA group (*p* < 0.01). And there was no obvious difference between the BCP + Sham EA group and BCP group (Figures 6C,D). The phosphorylation of mTOR Ser2481 was upregulated in the BCP, BCP + Sham EA, and BCP + EA groups compared with the control group (*p* < 0.05 or *p* < 0.01). However, the phosphorylation level between these three groups showed no obvious difference, indicating no significant regulative effect of EA (Figure 6E). As described, the phosphorylation of mTOR at Ser2448 and Thr2446 was consistent with the phosphoproteomic profiling data. In addition, the S6K and Akt total protein remained unchanged between different groups (Figures 7A–C). In comparison, the phosphorylation of S6K at Thr389, a marker of mTOR–raptor complex (mTORC1), and the phosphorylation of Akt at Ser473, a marker of mTOR–rictor complex (mTORC2), were also upregulated in the BCP and BCP + Sham EA groups compared with the control group, whereas downregulated by EA treatment in the BCP + EA group (*p* < 0.01). And they showed no significant change in the BCP + Sham EA group compared with the BCP group (Figures 7A,D,E).

### Inhibition of Mammalian Target of Rapamycin Signaling Alleviated the Mechanical Pain Behavior of Bone Cancer Pain Rats

**FIGURE 8:**
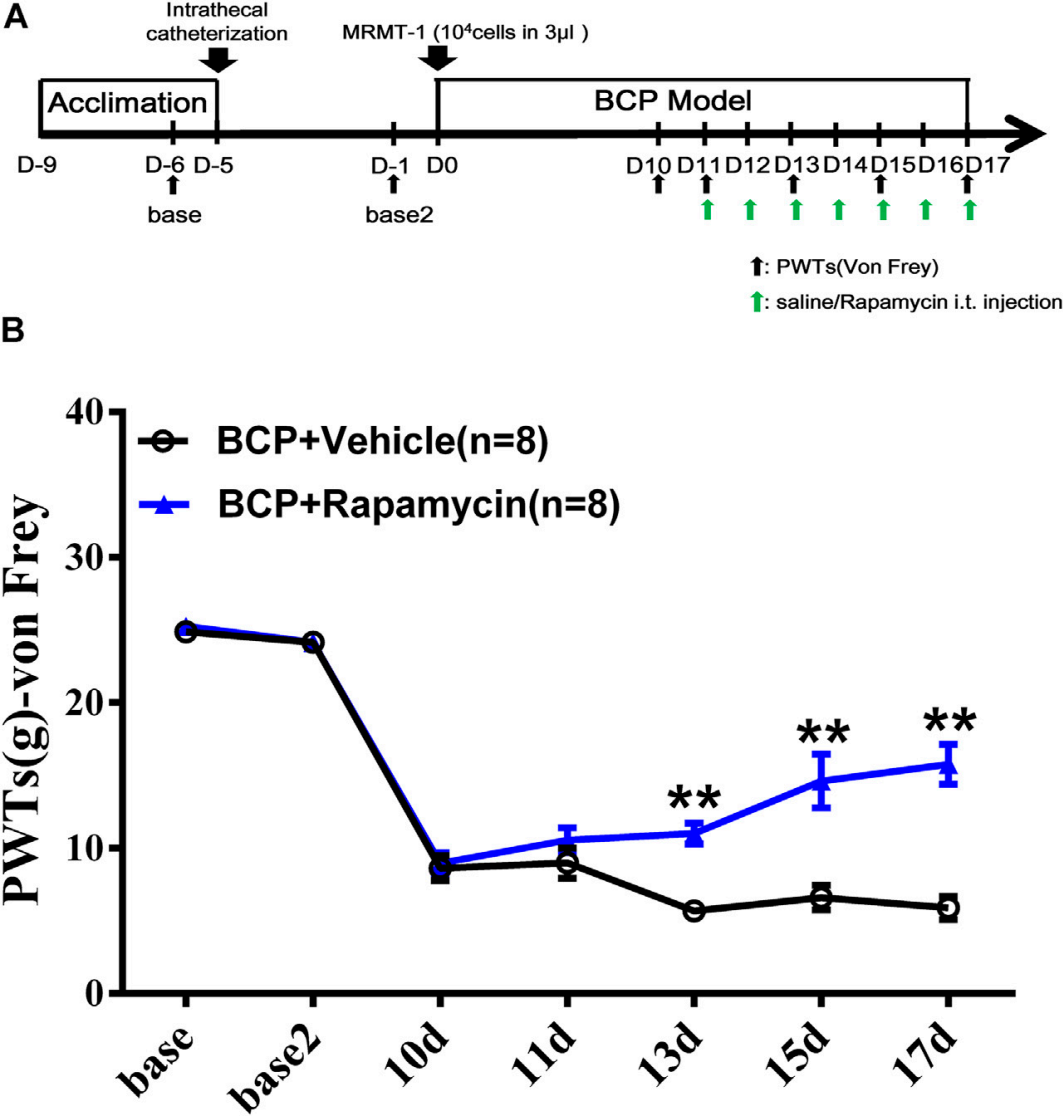
Administration of mTOR inhibitor rapamycin alleviated the mechanical hyperalgesia of BCP rats. **(A)** Animal experiment procedure for mTOR inhibition in BCP rats; **(B)** time-course change of PWTs in rats. The PWTs in the BCP + rapamycin group decreased on days 13, 15, and 17 after tumor cell inoculation. Compared with the BCP + vehicle group, the PWTs of BCP + rapamycin group increased on postoperative days 13, 15, and 17. **p < 0.01, compared with the BCP + vehicle group.

To clarify the role of mTOR signaling pathway in BCP, rapamycin, a selective inhibitor of mTOR, was used. The inhibitor was administered daily, intrathecally, starting from postoperative day 11 to day 17, with sterilized saline as vehicle (Figure 8A). As shown in Figure 8B, BCP-induced mechanical hyperalgesia was significantly attenuated by rapamycin on postinoculation days of 13, 15, and 17 (*p* < 0.01, compared with the BCP + vehicle group). This indicates that mTOR signaling is involved in the mechanism of mechanical hyperalgesia in BCP rats.

### The Administration of Mammalian Target of Rapamycin Agonist L-Leucine Impaired the Analgesic Effect of Electro-Acupuncture in Bone Cancer Pain Rats

**FIGURE 9:**
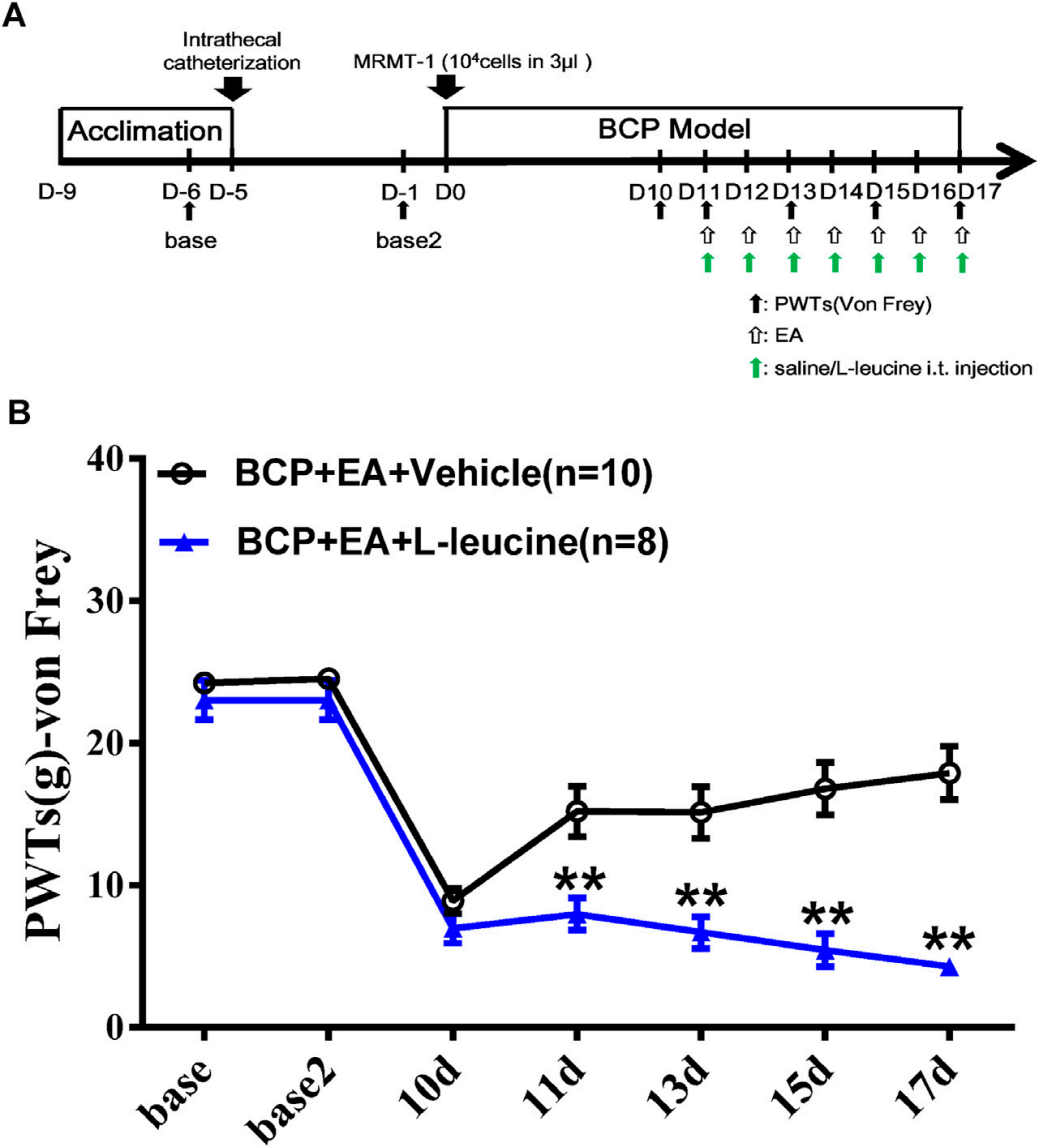
Administration of mTOR agonist L-leucine impaired the analgesic effect of EA in BCP rats. **(A)** Animal experiment procedure for mTOR agonism in EA-treated BCP rats; **(B)** time-course change of PWTs. Compared with the BCP + EA + vehicle group, the PWTs of the BCP + EA + L-leucine group dropped drastically on postoperative days 11, 13, 15, and 17. **p < 0.01, compared with the BCP + EA + vehicle group.

To explore whether mTOR signaling was involved in the analgesic effect of EA, a selective agonist of mTOR (L-leucine) was applied. The agonist was administered intrathecally 45 min before EA treatment, beginning from day 11 to day 17. The administration frequency was once per day, same as EA treatment (Figure 9A). As shown in Figure 9B, compared with EA treatment, the combination of EA treatment and administration of L-leucine significantly reduced the PWTs of rats on days 11, 13, 15, and 17 after carcinoma cell inoculation (*p* < 0.01, compared with the BCP + EA + vehicle group). This indicates that the analgesic effect of EA on BCP was impaired by mTOR agonist. In other words, mTOR signaling participates in the analgesic effect of EA.

## Discussion

In the present study, we established a rat model of BCP and verified the pain-like behavior by PWT measurement. Next, the phosphoproteomic profiling of the DRGs innervating the ipsilateral hind limb of BCP rats was detected by means of Phospho Explorer Antibody Array PEX100. DEPs were sorted out by the fold change of 1.6, and further underwent GO analysis and KEGG analysis to find enriched pathways from different classified aspects. Then the mTOR signaling pathway was targeted and expressions of mTOR, S6K, and Akt phosphorylation were confirmed by Western blot. Finally, the mTOR signaling inhibitor and agonist were applied to explore its role in BCP modeling and EA treatment. At present, the majority of studies focus on specific molecule, but little is known regarding the alterations in the phosphoproteomic profile in DRGs of BCP rats, especially in the field of EA analgesia (Largent-Milnes et al., 2008; Gaspari et al., 2017; Deng et al., 2019). Therefore, our experimental study filled the gap in profiling phosphoproteomic changes and key pathways in the primary sensory ganglions of EA-treated BCP rats by using Phospho Explorer Antibody Array PEX100.

The BCP modeling in this article referred to the classic and mature model of Medhurst et al. (2002). Consistent with previous research (Medhurst et al., 2002; Ducourneau et al., 2014; Zhu et al., 2017), the inoculation of MRMT-1 mammary gland carcinoma cells into the medullary cavity of tibia resulted in obvious mechanical hyperalgesia (the PWTs declined more than 50% than the baseline) since postoperative day 10 and continued to deteriorate in the following days until day 17. Those rats which did not show significant mechanical hyperalgesia were eliminated. The success rate of BCP modeling was reported as about 80% in this study. Consistent with our previous research, the BCP modeling rats also showed significant bone destruction and tumor growth (Du et al., 2016; Fu et al., 2017; Liang et al., 2018). After each intervention of EA treatment, the PWTs of BCP + EA rats were higher than those of BCP and BCP + Sham EA rats, but remained lower than those of control rats, indicating the efficacy of EA in BCP. Our group has also demonstrated the efficacy of EA with different frequency or treatment interval in treating BCP previously (Du et al., 2015; Liang et al., 2018). In this research, we identified that EA treatment attenuated the bone destruction of BCP rats to some extent, while sham EA treatment had no obvious effect.

According to GO analyses of the DEPs in DRGs, we discovered that the significantly enriched biological processes and molecular function of DEPs in BCP modeling or EA-treated rats were mostly involved in protein phosphorylation, response to drug, protein binding, ATP binding, protein kinase binding, and transcription factor binding. Apart from protein phosphorylation, positive regulation of transcription from RNA polymerase II promoter and negative regulation of apoptotic process were major biological processes of DEPs upregulated in the BCP and downregulated in the BCP + EA group; response to organic cyclic compound was main biological processes of DEPs downregulated in the BCP and upregulated in the BCP + EA group. These findings suggest that protein phosphorylation is likely to be a predominant process involved in the pathophysiology of the BCP model or EA treatment. The result coincided with previous studies showing that the phosphorylation of certain molecules in either central or peripheral nervous system was critically involved in hyperalgesia of BCP rats (Pareek et al., 2006; Hanamura et al., 2017). The phosphoproteomic profiling data in oxycodone-treated spinal cord also identified the importance of protein phosphorylation in the mechanism of BCP on a larger scale (Deng et al., 2019). The upregulated DEPs after BCP modeling participated in negative regulation of apoptotic process, preventing the apoptosis of cancer cells to some extent (Solary et al., 2000). The DEPs downregulated after BCP modeling and upregulated by EA contributed to the response to organic cyclic compound or drug, facilitating the response to drug therapy under cancer circumstance. From cellular component analysis, the most abundant component was cytoplasm, nucleus, cytosol, and nucleoplasm. We have also noticed the participation of neuron projection and axon, indicating the involvement of nervous system. The receptors of DRG primary afferent neurons can bind to a variety of cytokines released by inflammatory cells in the tumor environment to promote the sensitization of primary afferent neurons, resulting in pathological hyperplasia and the formation of neuroma, thus inducing peripheral sensitization of BCP (Nadler et al., 2000). The above findings demonstrated that protein phosphorylation and DRG neurobiological activity may be the potential mechanisms of EA-mediated analgesic effects in BCP.

For the 100 DEPs intersected by the BCP/Con and BCP + EA/BCP groups, it happens to be either upregulated in BCP and further downregulated by EA or downregulated in BCP and further upregulated by EA. KEGG analyses showed that the 100 DEPs were mainly involved in ErbB signaling pathway, MAPK signaling pathway, PI3K-Akt signaling pathway, apoptosis, AMPK (AMP-activated protein kinase) signaling pathway, mTOR signaling pathway, etc. Among these, ErbB signaling pathway and apoptosis were more related with cancer. In recent years, targeted therapy of ErbB2 has become a research hotspot in breast cancer treatment, and the upregulation of ErbB2 receptor was closely related to the occurrence of breast cancer (Xu et al., 2019). It is reported that MAPK and PI3K signaling pathways were involved in the upregulation of DRG TRPV1 expression under endogenous formaldehyde stimulation, thus playing an important role in BCP (Han et al., 2012). Activation of AMPK signaling pathway mediated spinal neuroinflammation or inflammatory responses for the attenuation of BCP (Han et al., 2012; Hao et al., 2020). In addition, many studies have already shown that MAPK and PI3K/Akt signaling pathways were involved in BCP mechanism from either the spinal cord or DRG level (Fang et al., 2015; Guan et al., 2015; Ni et al., 2019; Zhao et al., 2019). mTOR signaling pathway is the downstream of both ErbB and PI3K/Akt signaling pathway, and several studies have identified the importance of mTOR signaling pathway in the development and maintenance of BCP from the perspective of the central nervous system and *in vitro* research (Shih et al., 2012; Hsieh et al., 2015; Yuan et al., 2019). For example, BCP upregulation of pro-inflammatory cytokine signal in the periaqueductal gray amplified PI3K-mTOR signal in this brain region, contributing to BCP development (Jiang et al., 2016). PI3K-mTOR inhibitor PKI-402 suppressed breast cancer-induced osteoclast differentiation by impairing the PI3K-AKT-mTOR signaling pathway *in vitro* (Yuan et al., 2019). However, little research has been conducted to explore the effect of mTOR signaling pathway in BCP from DRG level, especially in EA area. In this research, the phosphorylation of mTOR (Ser2481, Ser2448, and Thr2446), S6K (Thr389), and Akt (Ser473) showed obvious opposite change in the BCP group and BCP + EA group, indicating that EA and BCP modeling has opposite effects on the phosphorylation of mTOR and its complex markers, thus mTOR may be a potential target of BCP under EA treatment.

EA has long been applied and identified to be useful in various types of clinical pain. Given that, EA was formally included in the NCCN cancer treatment guidelines for cancer pain treatment. In recent years, various studies have proved the efficacy of EA in BCP from animal experiments and clinical trials (Chen et al., 2008b; Paley et al., 2011; Ryu et al., 2014; Paley et al., 2015). As reported, EA at ST36 acupoint counteracted cancer pain, attenuated nociceptive effects in inflammatory pain conditions and also neuropathic pain (Zhang et al., 2012; Lu et al., 2018; Liu et al., 2019). Similar analgesic effects have also been observed in BL60 (Chang et al., 2012; Liang et al., 2016). Besides that, ST36 and BL60 were generally used in our previous research and had been identified to be effective in various pain conditions including BCP (Liang et al., 2018; Du et al., 2020). From our research, the Western blot result indicated that the expressions of p-mTOR (Ser2481, Ser2448, and Thr2446) were elevated in BCP rats, and EA can alleviate the mechanical pain of BCP rats through downregulation of p-mTOR (Ser2448 and Thr2446) but not p-mTOR (Ser2481). Although the p-mTOR (Ser2481) expression was contradicted, the expression trends of p-mTOR (Ser2448 and Thr2446) were coincident with phosphoproteomic profiling data. As reported, mTOR is the catalytic component of two distinct signaling complexes, mTOR–raptor complex (mTORC1) and mTOR–rictor complex (mTORC2). The molecular function of the two complexes remains poorly understood. Generally, mTORC1 is known to control protein synthesis, cell growth, cell proliferation, and cell cycle progression, whereas mTORC2 is associated with the control of actin cytoskeleton organization (Fingar and Blenis, 2004; Fingar et al., 2004; Jacinto et al., 2004; Kogasaka et al., 2013). The Ser2481 site of mTOR is an autophosphorylation site and is regarded as a marker of intact mTORC2 (Yonezawa et al., 2004; Copp et al., 2009). The Ser2448 phosphorylation site of mTOR is activated by S6K (p70) and occurs mainly in mTORC1, serving as a target of Akt (Yonezawa et al., 2004). Thr2446 is a novel mTOR phosphorylation site under nutrient-deprived state, where AMPKs were activated with a concomitant increase in phosphorylation of Thr2446. However, phosphorylation of Akt and the subsequent phosphorylation of Ser2448 were restricted when Thr2446 mutated to an acidic residue mimicking phosphorylation (Cheng et al., 2004). S6K phosphorylation at Thr389 is a well-established mTORC1-specific phosphorylation site and is generally used to evaluate the activity of S6K (Moustafa-Kamal et al., 2020; Napolitano et al., 2020). Akt Ser473 is also a marker of mTORC2, which is predominantly phosphorylated by mTORC2 as deleting any mTORC2 key components results in dramatic abrogation of this phosphorylation (Sarbassov et al., 2005; Jacinto et al., 2006). Their expressions were both increased in BCP rats and downregulated by EA treatment. Combined with our research, EA regulates both mTORC1 and mTORC2, directly or indirectly, affecting the process of protein synthesis and cell activities in BCP. This is consistent with findings in neuropathic pain evoked by spinal cord injury, in which mTORC1 inhibitor rapamycin alleviated mechanical and thermal hyperalgesia of model rats, and EA exhibited similar effect as rapamycin through downregulation of phosphorylation site on mTORC1 (Wang et al., 2019). Recently, selective inhibition of mTORC2 signaling by RNAi therapy showed being able to effectively block breast cancer cell growth and survival (Werfel et al., 2018). So mTORC2 might be a promising therapeutic target for cancer treatment. In some research, inhibition of mTORC1 and mTORC2 in the insular cortex attenuated neuropathic pain (Choi et al., 2020). However, the research on EA’s regulation of mTORC2 is scarce. In our research, the administration of mTOR inhibitor rapamycin alleviated the mechanical pain behavior of BCP rats, while mTOR agonist L-leucine impaired the analgesic effects of EA. These support that mTOR signaling pathway was involved in the development of BCP and EA analgesia in BCP. However, at this moment we do not know how mTORC1 and mTORC2 interact with each other in BCP or EA treatment and how exactly they are related to BCP or DRG functions and further studies are needed to verify this finding.

## Conclusion

So far, our study filled the gap in profiling phosphoproteomic analysis of protein phosphorylation and key pathways by using the PEX100 protein microarray in the primary sensory ganglia of the EA-treated BCP rat model. We identified mTOR phosphorylation and mTOR pathway may be potential approaches to relieve BCP by EA treatment. These findings may broaden our vision on the pain mechanisms of BCP, which in turn may contribute to explore a more therapeutic regimen for treating pain conditions associated with BCP.

## Data Availability

The datasets presented in this study can be found in online repositories. The names of the repository/repositories and accession number(s) can be found below: Protein Microarray Database [http://www.proteinmicroarray.cn/index.php?option=com_experiment&amp;view=detail&amp; experiment_id=256, PMDE256].

## Abbreviations

AMPK, AMP-activated protein kinase; BCP, Bone cancer pain; DEPs, differentially expressed phosphorylated proteins; DRGs, dorsal root ganglions; EA, electro-acupuncture; ErbB, erb-b2 receptor tyrosine kinase; ET, endothelin; GO, Gene Ontology; HRP, horseradish peroxidase; IARC, international agency for research on cancer; KEGG, Kyoto Encyclopedia of Genes and Genomes; MAPK, mitogen-activated protein kinase; mTOR, mammalian target of rapamycin; PG, prostaglandin; PI3K, phosphatidylinositol 3-kinase; PVDF, Polyvinylidene fluoride; PWTs, paw withdraw thresholds; TRPV1, transient receptor potential vanilloid-1.
