# Supplementary material for: Phosphoproteomic Profiling of Rat’s Dorsal Root Ganglia Reveals mTOR as a Potential Target in Bone Cancer Pain and Electro-Acupuncture’s Analgesia
Source: Front Pharmacol. 2021 Apr 29;12:593043. doi: 10.3389/fphar.2021.593043 (PMC8117331; doi:10.3389/fphar.2021.593043)
Supplement: Supplementary file 1 [file DataSheet1.docx]

Supplementary Material

# Supplementary Data

The phosphorylation changes of overall proteins and DEPs were shown in supplementary Table 1 and Table 2, respectively. The fold change of protein phosphorylation between groups and the corresponding *P* value were shown. However, in PEX100 Protein microarray assay, the fold change was majorly concerned in filtering DEPs.

The volcano plots were utilized to show the up-regulated and down-regulated phosphorylated proteins after BCP modeling or EA treatment (supplementary Figure 1). Taking *P* value into concern, the up-regulated DEPs were 6 after BCP modeling compared with the Control group and the down-regulated DEPs were 14. Compared with the BCP group, the up-regulated DEPs were 93 after EA treatment and the down-regulated DEPs were 10. The PEX100 Protein microarray assay we used was a high throughput technique for protein function analysis. In many high-quality studies, the same or the similar protein microarray technique was applied and the selection criterion of DEPs was the fold change but not the *P* value [1, 2]. So, the *P* value here was regarded as reference.

The phosphoproteomic proﬁle of mTOR detected by Phosphor-explorer array PEX100 assay was shown in heat map (supplementary Figure 2). As shown from the figure, the phosphorylation of mTOR Ser2448 was increased in BCP rats (*P* <0.05) and showed a downward trend after EA treatment. The phosphorylation of mTOR Thr2446 showed an upward trend after BCP modeling and a downward trend after EA treatment. However, the phosphorylation trend of mTOR (Ser2481) was totally the opposite, which was decreased in BCP rats (*P* <0.05) and up-regulated by EA (*P* <0.05).

# Supplementary Figures

**Supplementary Table 1.**

| Protein List | BCP/Con | BCP+EA/BCP | BCP+EA/Con |
| --- | --- | --- | --- |
| 14-3-3 beta/zeta (Phospho-Ser184/186) | 0.67* | 1.19 | 0.80 |
| 14-3-3 theta/tau (Phospho-Ser232) | 1.00 | 1.13 | 1.13 |
| 14-3-3 zeta (Phospho-Ser58) | 1.00 | 1.00 | 1.00 |
| 14-3-3 zeta/delta (Phospho-Thr232) | 1.04 | 1.28^▲^ | 1.33 |
| 4E-BP1 (Phospho-Ser65) | 0.39 | 4.93^▲▲^ | 1.92 |
| 4E-BP1 (Phospho-Thr36) | 2.02* | 0.64 | 1.30 |
| 4E-BP1 (Phospho-Thr45) | 1.26 | 0.82 | 1.03 |
| 4E-BP1 (Phospho-Thr70) | 0.92 | 1.02 | 0.95 |
| 6-phosphofructo-2-kinase/fructose-2,6-biphosphatase2 (PFKFB2) (Phospho-Ser483) | 0.91 | 1.14 | 1.04 |
| Abl1 (Phospho-Thr754/735) | 2.36 | 0.36 | 0.86 |
| Abl1 (Phospho-Tyr204) | 0.78 | 4.63 | 3.63 |
| ACC1 (Phospho-Ser79) | 0.99 | 0.99 | 0.98 |
| ACC1 (Phospho-Ser80) | 1.36 | 0.99 | 1.34 |
| Actin Pan (a/b/g) (Phospho-Tyr55/53) | 0.97 | 1.19 | 1.15 |
| ADD1 (Phospho-Ser726) | 0.89 | 1.53 | 1.36 |
| AFX/FOXO4 (Phospho-Ser197) | 0.59* | 2.27^▲^ | 1.33 |
| AKT1 (Phospho-Ser124) | 1.04 | 1.00 | 1.04 |
| AKT1 (Phospho-Ser246) | 0.92 | 1.16 | 1.07 |
| AKT1 (Phospho-Ser473) | 0.73 | 1.13 | 0.82 |
| AKT1 (Phospho-Thr308) | 0.80 | 1.29 | 1.04 |
| AKT1 (Phospho-Thr450) | 0.50 | 1.49 | 0.75 |
| AKT1 (Phospho-Thr72) | 1.04 | 1.24 | 1.29* |
| AKT1 (Phospho-Tyr326) | 0.39 | 4.17^▲^ | 1.64 |
| AKT1 (Phospho-Tyr474) | 0.64 | 2.93^▲▲^ | 1.87* |
| AKT1S1 (Phospho-Thr246) | 0.73 | 2.86^▲^ | 2.09* |
| AKT2 (Phospho-Ser474) | 1.27* | 0.76^▲▲^ | 0.97 |
| ALK (Phospho-Tyr1507) | 1.01 | 0.84 | 0.85 |
| ALK (Phospho-Tyr1604) | 1.61 | 0.90 | 1.44 |
| AMPK beta1 (Phospho-Ser182) | 0.96 | 1.41 | 1.34 |
| AMPK1 (Phospho-Thr172) | 1.25 | 0.75 | 0.94 |
| AMPK1/AMPK2 (Phospho-Ser485/491) | 0.86 | 1.46^▲^ | 1.26 |
| Amyloid beta A4 (Phospho-Thr743/668) | 0.73 | 1.51 | 1.11 |
| Androgen Receptor (Phospho-Ser213) | 0.88 | 0.95 | 0.84 |
| Androgen Receptor (Phospho-Ser650) | 0.58* | 1.94^▲^ | 1.13* |
| A-RAF (Phospho-Tyr301/302) | 0.76 | 1.20 | 0.91* |
| Arrestin-1 (Phospho-Ser412) | 0.77 | 1.67^▲▲▲^ | 1.29 |
| ASK1 (Phospho-Ser83) | 1.48 | 0.92 | 1.36 |
| ASK1 (Phospho-Ser966) | 1.09 | 0.99 | 1.08* |
| ATF1 (Phospho-Ser63) | 0.93* | 1.04 | 0.96 |
| ATF2 (Phospho-Ser112/94) | 1.18 | 0.90 | 1.06 |
| ATF2 (Phospho-Ser62/44) | 0.69 | 1.50^▲^ | 1.03 |
| ATF2 (Phospho-Thr69/51) | 0.85 | 1.13 | 0.96 |
| ATF2 (Phospho-Thr71/53) | 0.92 | 1.14 | 1.05 |
| ATF2 (Phospho-Thr73/55) | 1.14 | 0.90 | 1.03 |
| ATF4 (Phospho-Ser245) | 1.55 | 0.64 | 1.00 |
| ATP1A1/Na+K+ ATPase1 (Phospho-Ser23) | 0.80 | 1.35 | 1.08 |
| ATPase (Phospho-Ser16) | 1.11 | 1.17 | 1.29 |
| ATP-Citrate Lyase (Phospho-Ser454) | 0.74 | 1.43^▲^ | 1.06 |
| ATRIP (Phospho-Ser68/72) | 0.51 | 3.27^▲^ | 1.67 |
| AurA (Phospho-Ser342) | 1.28* | 0.92 | 1.17* |
| AurA (Phospho-Thr288) | 0.88 | 1.07 | 0.94 |
| AurB (Phospho-Thr232) | 1.22 | 0.99 | 1.21* |
| AurB (Phospho-Tyr12) | 1.23 | 0.82 | 1.01 |
| BAD (Phospho-Ser112) | 0.65* | 1.52 | 0.99 |
| BAD (Phospho-Ser134) | 0.88 | 1.44 | 1.26 |
| BAD (Phospho-Ser136) | 1.12 | 0.96 | 1.08 |
| BAD (Phospho-Ser155) | 0.62* | 2.15^▲▲^ | 1.33* |
| BAD (Phospho-Ser91/128) | 0.60** | 1.48^▲^ | 0.89* |
| BAX (Phospho-Thr167) | 0.77* | 1.31^▲^ | 1.01 |
| BCL-2 (Phospho-Ser70) | 1.61 | 1.00 | 1.62 |
| BCL-2 (Phospho-Thr56) | 1.33 | 0.96 | 1.28 |
| BCL-2 (Phospho-Thr69) | 1.06 | 0.88 | 0.94* |
| BCL-XL (Phospho-Thr47) | 0.56 | 3.21^▲^ | 1.80 |
| BCR (Phospho-Tyr177) | 1.22 | 1.11 | 1.35 |
| BCR (Phospho-Tyr360) | 1.19 | 0.61 | 0.72 |
| BID (Phospho-Ser78) | 0.78 | 2.56 | 1.99 |
| BIM (Phospho-Ser69/65) | 1.29 | 0.87 | 1.12 |
| BLNK (Phospho-Tyr96) | 0.68 | 4.15 | 2.84 |
| B-RAF (Phospho-Ser446) | 1.87 | 0.63 | 1.18 |
| B-RAF (Phospho-Ser601) | 1.06 | 0.91 | 0.97 |
| B-RAF (Phospho-Thr598) | 0.94 | 1.85 | 1.75 |
| BRCA1 (Phospho-Ser1457) | 0.76 | 1.59 | 1.20 |
| BRCA1 (Phospho-Ser1524) | 0.78* | 1.69^▲^ | 1.31 |
| BTK (Phospho-Tyr223) | 0.76 | 1.48 | 1.13 |
| c-Abl (Phospho-Tyr412) | 1.19 | 0.74 | 0.88 |
| Calmodulin (Phospho-Thr79/Ser81) | 0.78 | 1.95 | 1.51 |
| Calsenilin/KCNIP3 (Phospho-Ser63) | 0.71 | 1.15 | 0.81* |
| CaMK1-alpha (Phospho-Thr177) | 0.98 | 0.63^▲^ | 0.62 |
| CaMK2A (Phospho-Thr286) | 0.51* | 2.06^▲^ | 1.04 |
| CaMK2-beta/gamma/delta (Phospho-Thr287) | 0.87 | 1.27 | 1.11 |
| CaMK4 (Phospho-Thr196/200) | 0.96 | 0.97 | 0.94 |
| Caspase 1 (Phospho-Ser376) | 0.81 | 1.32 | 1.06 |
| Caspase 2 (Phospho-Ser157) | 1.22 | 0.96 | 1.17 |
| Caspase 3 (Phospho-Ser150) | 0.50 | 1.78 | 0.89 |
| Caspase 6 (Phospho-Ser257) | 0.71 | 1.46 | 1.04 |
| Caspase 8 (Phospho-Ser347) | 0.77 | 1.62 | 1.25 |
| Caspase 9 (Phospho-Ser144) | 0.97 | 1.11^▲^ | 1.07 |
| Caspase 9 (Phospho-Ser196) | 0.65 | 1.86 | 1.21 |
| Caspase 9 (Phospho-Thr125) | 0.34** | 2.19^▲^ | 0.75* |
| Caspase 9 (Phospho-Tyr153) | 0.88 | 1.04 | 0.92 |
| Catalase (Phospho-Tyr385) | 0.89 | 1.17 | 1.05 |
| Catenin beta (Phospho-Ser33) | 1.29 | 0.83 | 1.07* |
| Catenin beta (Phospho-Ser37) | 0.48 | 2.23 | 1.06 |
| Catenin beta (Phospho-Thr41/Ser45) | 0.85 | 1.13 | 0.96 |
| Catenin beta (Phospho-Tyr489) | 1.03 | 0.96 | 0.99 |
| Catenin beta (Phospho-Tyr654) | 0.51* | 1.79^▲^ | 0.91 |
| Catenin delta-1 (Phospho-Tyr228) | 1.21 | 0.80 | 0.97 |
| Caveolin-1 (Phospho-Tyr14) | 1.34* | 1.08 | 1.45* |
| CD19 (Phospho-Tyr531) | 0.64* | 1.45 | 0.93 |
| CD227/mucin 1 (Phospho-Tyr1243) | 1.05 | 0.92 | 0.97 |
| CD3Z (Phospho-Tyr142) | 0.99 | 1.56 | 1.54 |
| CD4 (Phospho-Ser433) | 0.83 | 1.47^▲^ | 1.21* |
| CD5 (Phospho-Tyr453) | 0.50* | 2.48^▲^ | 1.23** |
| CDC25A (Phospho-Ser124) | 1.60 | 0.52 | 0.83 |
| CDC25A (Phospho-Ser75) | 1.62 | 0.48 | 0.78 |
| CDC25B (Phospho-Ser323) | 0.98 | 1.03 | 1.00 |
| CDC25B (Phospho-Ser353) | 1.82* | 0.50^▲^ | 0.91 |
| CDC25C (Phospho-Ser216) | 0.31*** | 4.49^▲▲^ | 1.40* |
| CDK1/CDC2 (Phospho-Thr14) | 2.55*** | 0.44^▲^ | 1.13 |
| CDK1/CDC2 (Phospho-Tyr15) | 1.07 | 0.97 | 1.05 |
| CDK2 (Phospho-Thr160) | 1.14 | 0.66^▲^ | 0.75 |
| CDK5 (Phospho-Tyr15) | 0.85 | 1.54 | 1.31 |
| CDK7 (Phospho-Thr170) | 0.91 | 1.15 | 1.05 |
| Chk1 (Phospho-Ser280) | 1.20 | 0.78^▲^ | 0.94 |
| Chk1 (Phospho-Ser286) | 0.69 | 1.94^▲▲^ | 1.34* |
| Chk1 (Phospho-Ser317) | 0.86 | 1.87 | 1.60* |
| Chk1 (Phospho-Ser345) | 1.34 | 0.93 | 1.24 |
| Chk2 (Phospho-Ser516) | 0.68 | 1.64 | 1.11 |
| Chk2 (Phospho-Thr383) | 0.67 | 1.91 | 1.29* |
| Chk2 (Phospho-Thr387) | 0.87 | 1.55 | 1.35 |
| Chk2 (Phospho-Thr68) | 0.85 | 1.59^▲^ | 1.35* |
| c-Jun (Phospho-Ser243) | 1.41 | 0.70 | 0.98 |
| c-Jun (Phospho-Ser63) | 1.07* | 1.10 | 1.18 |
| c-Jun (Phospho-Thr239) | 0.89 | 1.18 | 1.04 |
| c-Jun (Phospho-Thr91) | 1.53** | 0.68 | 1.04 |
| c-Jun (Phospho-Thr93) | 1.63 | 0.60 | 0.99 |
| c-Jun (Phospho-Tyr170) | 0.64 | 1.72^▲^ | 1.11 |
| CK1-A (Phospho-Thr321) | 1.20 | 0.59 | 0.71 |
| CK2-b (Phospho-Ser209) | 1.37* | 0.71^▲^ | 0.97 |
| Claudin 3 (Phospho-Tyr219) | 0.90 | 1.06 | 0.95 |
| Claudin 7 (Phospho-Tyr210) | 0.88 | 0.96 | 0.85 |
| Cofilin (Phospho-Ser3) | 0.86 | 1.26 | 1.09 |
| Connexin 43 (Phospho-Ser367) | 1.11 | 1.30 | 1.45* |
| Cortactin (Phospho-Tyr421) | 1.03 | 1.09 | 1.12 |
| Cortactin (Phospho-Tyr466) | 0.90 | 1.45^▲^ | 1.30* |
| CPI17 alpha (Phospho-Thr38) | 0.73* | 1.50 | 1.10 |
| c-PLA2 (Phospho-Ser505) | 1.61** | 0.79^▲^ | 1.27* |
| CREB (Phospho-Ser121) | 1.13 | 0.92 | 1.04 |
| CREB (Phospho-Ser129) | 0.71** | 1.49^▲▲^ | 1.06 |
| CREB (Phospho-Ser133) | 2.13** | 0.59^▲▲^ | 1.25** |
| CREB (Phospho-Ser142) | 0.86 | 1.16 | 1.00 |
| CREB (Phospho-Thr100) | 1.43 | 0.64 | 0.91 |
| CrkII (Phospho-Tyr221) | 1.15 | 0.86 | 0.99 |
| Cyclin B1 (Phospho-Ser126) | 1.08 | 0.95 | 1.03 |
| Cyclin B1 (Phospho-Ser147) | 0.82 | 1.10 | 0.90 |
| Cyclin D1 (Phospho-Thr286) | 1.04 | 2.06 | 2.14 |
| Cyclin D3 (Phospho-Thr283) | 0.85 | 1.12 | 0.95 |
| Cyclin E1 (Phospho-Thr395) | 0.76* | 0.87 | 0.66* |
| Cyclin E1 (Phospho-Thr77) | 0.52** | 4.09 | 2.13 |
| Cyclin E2 (Phospho-Thr392) | 0.71* | 1.73^▲▲^ | 1.23 |
| DAB1 (Phospho-Tyr220) | 1.12 | 1.05 | 1.18* |
| DAB1 (Phospho-Tyr232) | 0.58* | 1.99^▲^ | 1.14 |
| DAPP1 (Phospho-Tyr139) | 0.74 | 1.92^▲^ | 1.42 |
| DARPP-32 (Phospho-Thr34) | 0.81 | 1.14 | 0.93 |
| DARPP-32 (Phospho-Thr75) | 1.14 | 0.88 | 1.00 |
| DDX5/DEAD-box protein 5 (Phospho-Tyr593) | 0.88 | 1.18 | 1.04 |
| DNA-PK (Phospho-Thr2638) | 1.04 | 0.88 | 0.92 |
| DNA-PK (Phospho-Thr2647) | 2.13 | 0.35 | 0.74* |
| Dok-1 (Phospho-Tyr362) | 0.70 | 1.70^▲^ | 1.19 |
| Dok-1 (Phospho-Tyr398) | 0.55* | 1.92^▲^ | 1.06 |
| Dok-2 (Phospho-Tyr299) | 0.96 | 0.91 | 0.87* |
| DYN1 (Phospho-Ser774) | 0.51 | 3.21 | 1.63 |
| E2F1 (Phospho-Thr433) | 0.54* | 2.17 | 1.17 |
| EEF2 (Phospho-Thr56) | 0.75 | 2.03^▲^ | 1.52* |
| eEF2K (Phospho-Ser366) | 1.15 | 0.91 | 1.04 |
| EGFR (Phospho-Ser1070) | 0.53* | 2.50^▲^ | 1.33 |
| EGFR (Phospho-Thr678) | 1.08 | 0.87 | 0.94 |
| EGFR (Phospho-Thr693) | 0.65* | 1.54^▲^ | 1.00 |
| EGFR (Phospho-Tyr1016) | 1.03 | 1.24 | 1.28 |
| EGFR (Phospho-Tyr1069) | 1.37 | 0.71 | 0.98 |
| EGFR (Phospho-Tyr1092) | 1.06 | 1.19 | 1.26 |
| EGFR (Phospho-Tyr1110) | 1.06 | 1.12 | 1.19 |
| EGFR (Phospho-Tyr1172) | 0.66* | 1.82^▲^ | 1.20 |
| EGFR (Phospho-Tyr1197) | 0.81 | 1.23 | 1.00 |
| EGFR (Phospho-Tyr869) | 0.67* | 2.36^▲^ | 1.58 |
| eIF2A (Phospho-Ser51) | 1.35* | 1.05 | 1.41 |
| eIF4E (Phospho-Ser209) | 0.79 | 1.20 | 0.95 |
| eIF4G (Phospho-Ser1108) | 0.79 | 1.96 | 1.55* |
| Elk1 (Phospho-Ser383) | 0.91 | 1.08 | 0.99 |
| Elk1 (Phospho-Ser389) | 0.41* | 2.74^▲^ | 1.12* |
| Elk1 (Phospho-Thr417) | 1.25 | 0.86 | 1.07 |
| eNOS (Phospho-Ser1177) | 0.62* | 1.52 | 0.94 |
| eNOS (Phospho-Ser615) | 1.29 | 0.73 | 0.94 |
| eNOS (Phospho-Thr495) | 1.03 | 0.95 | 0.98 |
| EPB41 (Phospho-Tyr418/660) | 1.09 | 0.90 | 0.99 |
| EPHA2/3/4 (Phospho-Tyr588/596) | 0.79 | 1.59 | 1.27 |
| EPHB1/2 (Phospho-Tyr594/604) | 1.22 | 0.87 | 1.06 |
| Ephrin B1 (Phospho-Tyr317) | 1.32 | 0.77 | 1.02 |
| Ephrin B2 (Phospho-Tyr330) | 0.54* | 2.35^▲^ | 1.26 |
| Epo-R (Phospho-Tyr368) | 0.82 | 1.43 | 1.17 |
| ERK3 (Phospho-Ser189) | 0.41 | 1.38^▲^ | 0.56 |
| Estrogen Receptor-alpha (Phospho-Ser104) | 0.58 | 2.01 | 1.17 |
| Estrogen Receptor-alpha (Phospho-Ser106) | 0.70 | 2.58^▲^ | 1.80* |
| Estrogen Receptor-alpha (Phospho-Ser118) | 0.98 | 0.99 | 0.97 |
| Estrogen Receptor-alpha (Phospho-Ser167) | 1.14 | 0.81 | 0.93 |
| ETK (Phospho-Tyr40) | 0.61 | 1.74 | 1.06 |
| Ezrin (Phospho-Thr566) | 0.36* | 3.88^▲^ | 1.40* |
| Ezrin (Phospho-Tyr353) | 1.09 | 0.81 | 0.88 |
| Ezrin (Phospho-Tyr478) | 0.72* | 1.47 | 1.06 |
| FAK (Phospho-Ser910) | 0.70 | 3.20^▲^ | 2.23* |
| FAK (Phospho-Tyr397) | 0.46 | 2.29 | 1.05 |
| FAK (Phospho-Tyr407) | 0.95 | 1.08 | 1.02 |
| FAK (Phospho-Tyr576) | 1.04 | 0.91 | 0.95 |
| FAK (Phospho-Tyr861) | 1.35 | 0.93 | 1.25 |
| FAK (Phospho-Tyr925) | 1.07 | 1.03 | 1.11* |
| FAS (Phospho-Tyr291) | 1.19 | 1.64 | 1.95 |
| FER (Phospho-Tyr402) | 0.59 | 1.92 | 1.12 |
| FGFR1 (Phospho-Tyr154) | 1.41 | 0.78 | 1.10 |
| FGFR1 (Phospho-Tyr766) | 0.99 | 1.24 | 1.22 |
| Filamin A (Phospho-Ser2152) | 0.81* | 1.45^▲^ | 1.18** |
| FKHR (Phospho-Ser256) | 0.92 | 0.93 | 0.85 |
| FKHR (Phospho-Ser319) | 1.18 | 1.75 | 2.06* |
| FKHR/FOXO1A (Phospho-Ser329) | 0.87 | 1.15^▲▲^ | 1.01 |
| FKHRL1/FOXO3A (Phospho-Ser253) | 0.87 | 2.26 | 1.96* |
| FLT3 (Phospho-Tyr599) | 1.43 | 0.82 | 1.18 |
| Fos (Phospho-Thr232) | 0.63 | 1.28 | 0.81 |
| FosB (Phospho-Ser27) | 0.75 | 1.36 | 1.02 |
| FOXO1/3/4-pan (Phospho-Thr24/32) | 0.94 | 1.28 | 1.21 |
| G3BP-1 (Phospho-Ser232) | 0.98 | 1.16 | 1.13 |
| Gab1 (Phospho-Tyr627) | 1.05 | 1.08 | 1.14** |
| Gab1 (Phospho-Tyr659) | 1.26 | 0.86 | 1.09 |
| Gab2 (Phospho-Ser159) | 1.16 | 0.58^▲^ | 0.67 |
| GABA-RB (Phospho-Ser434) | 1.13 | 0.80^▲^ | 0.91 |
| GAP43 (Phospho-Ser41) | 0.79 | 1.43 | 1.12 |
| GATA1 (Phospho-Ser142) | 0.83* | 1.06 | 0.88 |
| GATA1 (Phospho-Ser310) | 0.99 | 1.08 | 1.07 |
| GluR1 (Phospho-Ser849) | 0.42* | 7.28^▲^ | 3.05 |
| GluR1 (Phospho-Ser863) | 1.02 | 1.67 | 1.70 |
| GluR2 (Phospho-Ser880) | 0.65 | 3.26 | 2.13 |
| GRB10/Growth factor receptor-bound protein 10 (Phospho-Tyr67) | 0.88* | 1.02 | 0.90 |
| GRK1 (Phospho-Ser21) | 1.27 | 0.86 | 1.09 |
| GRK2 (Phospho-Ser29) | 0.64 | 2.61^▲▲^ | 1.68 |
| GSK3 alpha (Phospho-Ser21) | 1.02 | 0.87 | 0.89 |
| GSK3 alpha/beta (Phospho-Tyr216/279) | 0.81 | 1.15 | 0.93 |
| GSK3 beta (Phospho-Ser9) | 1.41 | 0.68 | 0.96 |
| GTPase activating protein (Phospho-Ser387) | 0.83 | 1.26^▲^ | 1.04 |
| HDAC1 (Phospho-Ser421) | 1.26 | 5.79^▲▲^ | 7.31* |
| HDAC2 (Phospho-Ser394) | 1.62** | 0.56^▲^ | 0.92 |
| HDAC3 (Phospho-Ser424) | 0.76 | 1.91^▲▲^ | 1.45* |
| HDAC4 (Phospho-Ser632) | 1.48* | 0.75 | 1.11 |
| HDAC5 (Phospho-Ser259) | 0.83 | 1.19 | 0.99 |
| HDAC5 (Phospho-Ser498) | 0.81 | 1.31 | 1.06 |
| HDAC6 (Phospho-Ser22) | 0.46* | 2.65^▲^ | 1.22 |
| HDAC8 (Phospho-Ser39) | 1.44 | 1.09 | 1.57** |
| HER2 (Phospho-Thr686) | 1.03 | 1.01 | 1.04 |
| HER2 (Phospho-Tyr1221/Tyr1222) | 1.04 | 1.60^▲^ | 1.66 |
| HER2 (Phospho-Tyr1248) | 1.08 | 1.06 | 1.14 |
| HER2 (Phospho-Tyr877) | 1.73 | 0.67 | 1.16 |
| HER3/ErbB3 (Phospho-Tyr1222) | 1.17 | 0.59^▲^ | 0.69 |
| HER3/ErbB3 (Phospho-Tyr1289) | 0.93 | 0.99 | 0.92 |
| HER4/ErbB4 (Phospho-Tyr1284) | 0.62 | 1.85 | 1.14 |
| Histone H2A.X (Phospho-Ser139) | 0.60 | 1.74 | 1.04 |
| Histone H3.1 (Phospho-Ser10) | 0.83 | 1.93^▲^ | 1.61 |
| HNF4 alpha (Phospho-Ser313) | 0.82 | 1.11 | 0.91 |
| HRS (Phospho-Tyr334) | 1.22 | 0.66^▲^ | 0.81 |
| HSF1 (Phospho-Ser303) | 1.13 | 1.08 | 1.22 |
| HSL (Phospho-Ser552/563) | 0.68 | 1.37 | 0.93 |
| HSL (Phospho-Ser554) | 1.04 | 1.19 | 1.24 |
| HSP27 (Phospho-Ser15) | 1.00 | 0.97 | 0.97 |
| HSP27 (Phospho-Ser78) | 0.89 | 1.31^▲^ | 1.17 |
| HSP27 (Phospho-Ser82) | 1.01 | 0.96 | 0.97 |
| HSP90 co-chaperone Cdc37 (Phospho-Ser13) | 1.38* | 0.73^▲^ | 1.01 |
| HSP90B (Phospho-Ser226) | 0.75 | 1.01 | 0.75 |
| HSP90B (Phospho-Ser254) | 0.55 | 1.85^▲^ | 1.02 |
| ICAM-1 (Phospho-Tyr512) | 1.50 | 0.72 | 1.09 |
| IGF1R (Phospho-Tyr1161) | 1.83** | 0.60 | 1.10 |
| IGF1R (Phospho-Tyr1165/1166) | 1.37 | 0.53 | 0.73 |
| IGF2R (Phospho-Ser2409) | 0.69 | 1.48 | 1.03 |
| IkB-alpha (Phospho-Ser32/36) | 1.26 | 0.68 | 0.87 |
| IkB-alpha (Phospho-Tyr42) | 0.81 | 1.14 | 0.92 |
| IkB-beta (Phospho-Thr19) | 1.39 | 0.77^▲^ | 1.07 |
| IkB-epsilon (Phospho-Ser22) | 1.43 | 0.91 | 1.29 |
| IKK-alpha (Phospho-Thr23) | 0.77 | 1.47 | 1.13 |
| IKK-alpha/beta (Phospho-Ser180/181) | 0.81 | 1.51^▲^ | 1.23 |
| IKK-beta (Phospho-Tyr188) | 0.77 | 2.04^▲^ | 1.57 |
| IKK-beta (Phospho-Tyr199) | 0.51 | 2.57^▲▲▲^ | 1.30 |
| IKK-gamma (Phospho-Ser31) | 0.75 | 1.65 | 1.25 |
| IKK-gamma (Phospho-Ser85) | 1.55 | 1.04 | 1.61 |
| IL-10R-alpha (Phospho-Tyr496) | 1.36 | 0.84^▲^ | 1.14 |
| IL-13R/CD213a1 (Phospho-Tyr405) | 1.02 | 0.93 | 0.95 |
| IL-2RA/CD25 (Phospho-Ser268) | 0.93 | 1.09 | 1.01 |
| IL3RB (Phospho-Tyr593) | 1.11 | 0.97 | 1.08 |
| IL-4R/CD124 (Phospho-Tyr497) | 0.95 | 0.85 | 0.80* |
| Integrin beta-1 (Phospho-Thr788) | 1.27 | 0.81 | 1.03 |
| Integrin beta-3 (Phospho-Tyr773) | 1.04 | 0.90 | 0.93 |
| Integrin beta-3 (Phospho-Tyr785) | 1.04 | 1.12 | 1.16 |
| Integrin beta-4 (Phospho-Tyr1510) | 1.40* | 0.73^▲^ | 1.02 |
| Interferon-alpha/beta receptor alpha chain (Phospho-Tyr466) | 0.70 | 2.51^▲▲^ | 1.76* |
| Interferon-gamma receptor alpha chain precursor (Phospho-Tyr457) | 1.25 | 0.75 | 0.94 |
| IR (Phospho-Tyr1361) | 1.12 | 0.93 | 1.04 |
| IRS-1 (Phospho-Ser307) | 0.54** | 1.77^▲^ | 0.96 |
| IRS-1 (Phospho-Ser312) | 1.02 | 0.88 | 0.90 |
| IRS-1 (Phospho-Ser323) | 0.51 | 2.38^▲▲^ | 1.22 |
| IRS-1 (Phospho-Ser636) | 1.25 | 1.04 | 1.30* |
| IRS-1 (Phospho-Ser639) | 0.32* | 3.29^▲^ | 1.07 |
| IRS-1 (Phospho-Ser794) | 0.74 | 1.76 | 1.30 |
| JAK1 (Phospho-Tyr1022) | 0.72 | 1.67 | 1.20* |
| JAK2 (Phospho-Tyr1007) | 0.62 | 2.10 | 1.31 |
| JAK2 (Phospho-Tyr221) | 0.60* | 1.89^▲^ | 1.13 |
| JNK1/2/3 (Phospho-Thr183/Tyr185) | 1.34 | 0.82 | 1.10 |
| JunB (Phospho-Ser259) | 1.07 | 1.10 | 1.18 |
| JunB (Phospho-Ser79) | 1.88 | 0.76 | 1.42 |
| JunD (Phospho-Ser255) | 1.11 | 1.19 | 1.32 |
| Keratin 18 (Phospho-Ser33) | 0.56* | 1.46^▲^ | 0.82 |
| Keratin 18 (Phospho-Ser52) | 0.99 | 1.39 | 1.38 |
| Keratin 8 (Phospho-Ser431) | 1.98 | 0.71 | 1.40 |
| Keratin 8 (Phospho-Ser73) | 0.65* | 1.60^▲^ | 1.04 |
| KIT (Phospho-Tyr721) | 1.33 | 0.95 | 1.26 |
| KIT (Phospho-Tyr936) | 1.13 | 0.65 | 0.74 |
| KSR (Phospho-Ser392) | 0.94 | 0.99 | 0.94 |
| Kv1.3/KCNA3 (Phospho-Tyr135) | 1.05 | 0.79 | 0.83 |
| Lamin A (Phospho-Ser22) | 1.05 | 0.80 | 0.84 |
| Lamin A/C (Phospho-Ser392) | 0.39* | 4.79^▲▲^ | 1.85** |
| LAT (Phospho-Tyr171) | 0.59 | 1.84 | 1.08 |
| LAT (Phospho-Tyr191) | 0.60* | 1.72^▲^ | 1.03 |
| LCK (Phospho-Ser59) | 0.95 | 1.10 | 1.04 |
| LCK (Phospho-Tyr192) | 0.67 | 1.88^▲^ | 1.25 |
| LCK (Phospho-Tyr393) | 0.85** | 0.99 | 0.85** |
| LCK (Phospho-Tyr504) | 1.00 | 1.14 | 1.14* |
| LIMK1 (Phospho-Thr508) | 0.74 | 1.55 | 1.15 |
| LKB1 (Phospho-Ser428) | 0.89 | 1.10 | 0.98 |
| LKB1 (Phospho-Thr189) | 0.77 | 1.47^▲^ | 1.13 |
| LYN (Phospho-Tyr507) | 0.17 | 6.06 | 1.00 |
| MAP3K7/TAK1 (Phospho-Thr184) | 1.17 | 1.03 | 1.20* |
| MAP3K8/COT (Phospho-Thr290) | 0.59* | 2.06^▲▲^ | 1.22* |
| MAPKAPK2 (Phospho-Ser272) | 1.11 | 1.57 | 1.73 |
| MARCKS (Phospho-Ser158) | 0.83 | 1.12 | 0.93 |
| MARCKS (Phospho-Ser163) | 1.07 | 0.77 | 0.83 |
| M-CSF Receptor (Phospho-Tyr561) | 0.97 | 0.97 | 0.94 |
| M-CSF Receptor (Phospho-Tyr809) | 1.07 | 0.76 | 0.81 |
| MDM2 (Phospho-Ser166) | 0.78 | 2.36 | 1.84 |
| MEF2A (Phospho-Ser408) | 0.62* | 1.93^▲^ | 1.20 |
| MEF2A (Phospho-Thr312) | 0.78 | 1.79 | 1.40 |
| MEF2A (Phospho-Thr319) | 0.95* | 1.24 | 1.17 |
| MEF2C (Phospho-Ser396) | 0.95 | 1.24 | 1.18 |
| MEK1 (Phospho-Ser217) | 1.01 | 1.34 | 1.35 |
| MEK1 (Phospho-Ser221) | 1.16 | 0.74 | 0.86 |
| MEK1 (Phospho-Ser298) | 0.25* | 4.02 | 1.01 |
| MEK1 (Phospho-Thr286) | 1.34 | 0.79 | 1.07 |
| MEK1 (Phospho-Thr291) | 0.92 | 1.14 | 1.05 |
| MEK2 (Phospho-Thr394) | 1.44** | 0.67^▲▲▲^ | 0.96 |
| Merlin (Phospho-Ser10) | 0.43 | 6.15^▲^ | 2.64* |
| Merlin (Phospho-Ser518) | 1.28 | 0.99 | 1.27* |
| Met (Phospho-Tyr1003) | 0.49 | 2.11 | 1.03 |
| Met (Phospho-Tyr1234) | 1.28 | 0.94 | 1.19 |
| Met (Phospho-Tyr1349) | 0.67 | 1.45 | 0.97 |
| MITF (Phospho-Ser73) | 0.74 | 1.52 | 1.12 |
| MKK3/MAP2K3 (Phospho-Ser189) | 0.58* | 1.72^▲^ | 1.00 |
| MKK3/MAP2K3 (Phospho-Thr222) | 1.16 | 0.99 | 1.14 |
| MKK4/SEK1 (Phospho-Ser257) | 1.30 | 0.69^▲^ | 0.91 |
| MKK4/SEK1 (Phospho-Ser80) | 0.76 | 2.09^▲▲^ | 1.60 |
| MKK4/SEK1 (Phospho-Thr261) | 0.72 | 2.75^▲^ | 1.97 |
| MKK6/MAP2K6 (Phospho-Ser207) | 0.68* | 1.75^▲^ | 1.19 |
| MKK7/MAP2K7 (Phospho-Ser271) | 0.58 | 1.93^▲^ | 1.12 |
| MKP-1 (Phospho-Ser359) | 0.88 | 1.45 | 1.28** |
| MKP-1/2 (Phospho-Ser296/318) | 0.95 | 1.38 | 1.32* |
| Mnk1 (Phospho-Thr385) | 0.46* | 1.92^▲^ | 0.88 |
| MSK1 (Phospho-Ser360) | 0.98 | 1.20 | 1.18 |
| MSK1 (Phospho-Ser376) | 1.24 | 0.94 | 1.16 |
| MSK1 (Phospho-Thr581) | 1.13 | 0.90 | 1.02 |
| Mst1/Mst2 (Phospho-Thr183) | 1.08 | 0.91 | 0.98 |
| mTOR (Phospho-Ser2448) | 1.43* | 0.86 | 1.23 |
| mTOR (Phospho-Ser2481) | 0.41* | 2.31^▲^ | 0.96 |
| mTOR (Phospho-Thr2446) | 1.20 | 0.85 | 1.02 |
| Myc (Phospho-Ser373) | 0.86 | 1.39^▲^ | 1.20 |
| Myc (Phospho-Ser62) | 1.10 | 0.85 | 0.93 |
| Myc (Phospho-Thr358) | 0.69 | 2.55 | 1.76 |
| Myc (Phospho-Thr58) | 1.32 | 0.94 | 1.24** |
| Myosin regulatory light chain 2 (Phospho-Ser18) | 0.88 | 1.16 | 1.02 |
| NFAT4 (Phospho-Ser165) | 1.28 | 0.86 | 1.09 |
| NFkB-p100/p52 (Phospho-Ser865) | 0.97 | 1.38 | 1.35 |
| NFkB-p100/p52 (Phospho-Ser869) | 1.15 | 1.18 | 1.35 |
| NFkB-p105/p50 (Phospho-Ser337) | 1.08 | 0.82^▲^ | 0.89 |
| NFkB-p105/p50 (Phospho-Ser893) | 0.79 | 1.34 | 1.06 |
| NFkB-p105/p50 (Phospho-Ser907) | 1.07 | 1.03 | 1.10 |
| NFkB-p105/p50 (Phospho-Ser927) | 0.59 | 3.55 | 2.08 |
| NFkB-p105/p50 (Phospho-Ser932) | 0.94 | 1.04 | 0.98 |
| NFkB-p65 (Phospho-Ser276) | 1.12 | 0.91 | 1.02 |
| NFkB-p65 (Phospho-Ser311) | 0.45* | 2.44^▲▲^ | 1.09 |
| NFkB-p65 (Phospho-Ser468) | 1.86 | 0.48 | 0.90 |
| NFkB-p65 (Phospho-Ser529) | 1.33 | 0.99 | 1.31 |
| NFkB-p65 (Phospho-Ser536) | 1.22 | 1.55 | 1.89* |
| NFkB-p65 (Phospho-Thr254) | 0.92 | 1.19 | 1.09 |
| NFkB-p65 (Phospho-Thr435) | 1.25** | 0.91^▲^ | 1.14* |
| NFkB-p65 (Phospho-Thr505) | 0.77 | 1.46 | 1.13 |
| NMDAR1 (Phospho-Ser897) | 1.45 | 0.84 | 1.22 |
| NMDAR2B (Phospho-Tyr1472) | 0.58 | 2.83^▲^ | 1.64 |
| Opioid Receptor (Phospho-Ser375) | 1.65 | 0.53^▲^ | 0.87* |
| p130Cas (Phospho-Tyr165) | 0.75 | 1.61 | 1.21 |
| p130Cas (Phospho-Tyr410) | 0.61* | 1.42 | 0.86 |
| p21Cip1 (Phospho-Thr145) | 1.59 | 0.77 | 1.22 |
| p27Kip1 (Phospho-Ser10) | 1.17 | 1.00 | 1.17 |
| p27Kip1 (Phospho-Thr187) | 1.40 | 0.69^▲^ | 0.96 |
| p38 MAPK (Phospho-Thr180) | 1.38 | 0.71 | 0.98 |
| p38 MAPK (Phospho-Tyr182) | 0.54 | 3.20^▲▲^ | 1.72 |
| p38 MAPK (Phospho-Tyr322) | 0.88 | 0.89^▲^ | 0.78 |
| p44/42 MAPK (Phospho-Thr202) | 0.98 | 0.94 | 0.93 |
| p44/42 MAPK (Phospho-Tyr204) | 1.33 | 0.68 | 0.91 |
| p53 (Phospho-Ser15) | 0.87 | 1.34^▲^ | 1.16 |
| p53 (Phospho-Ser20) | 0.73 | 2.24^▲^ | 1.64* |
| p53 (Phospho-Ser315) | 1.01 | 0.80 | 0.81 |
| p53 (Phospho-Ser33) | 0.60 | 1.98 | 1.18 |
| p53 (Phospho-Ser37) | 0.86 | 0.98 | 0.83 |
| p53 (Phospho-Ser378) | 1.11 | 0.68 | 0.76 |
| p53 (Phospho-Ser392) | 1.20* | 1.03 | 1.24* |
| p53 (Phospho-Ser46) | 0.87 | 1.12 | 0.98 |
| p53 (Phospho-Ser6) | 1.32 | 0.91 | 1.20* |
| p53 (Phospho-Ser9) | 0.86 | 1.20^▲^ | 1.04 |
| p53 (Phospho-Thr18) | 1.00 | 0.97 | 0.96 |
| P70S6K (Phospho-Ser371) | 1.22* | 0.81^▲^ | 1.00 |
| P70S6K (Phospho-Ser411) | 0.60* | 2.01^▲^ | 1.20 |
| P70S6K (Phospho-Ser418) | 1.39* | 0.90 | 1.25 |
| P70S6K (Phospho-Ser424) | 1.09 | 0.98 | 1.07 |
| P70S6K (Phospho-Thr229) | 1.10 | 1.06 | 1.17 |
| P70S6K (Phospho-Thr421) | 1.30 | 1.00 | 1.29* |
| P70S6K-beta (Phospho-Ser423) | 0.90 | 1.26 | 1.14 |
| P73 (Phospho-Tyr99) | 0.68 | 1.59 | 1.09 |
| P90RSK (Phospho-Ser380) | 0.94 | 1.13 | 1.06 |
| P90RSK (Phospho-Thr359/Ser363) | 1.94 | 1.19 | 2.31* |
| P90RSK (Phospho-Thr573) | 1.16 | 1.48 | 1.72 |
| P95/NBS1 (Phospho-Ser343) | 0.74 | 2.16 | 1.59 |
| PAK1 (Phospho-Ser204) | 1.32 | 0.70 | 0.92 |
| PAK1 (Phospho-Thr212) | 0.97 | 0.87 | 0.85 |
| PAK1/2 (Phospho-Ser199) | 0.56 | 1.91 | 1.08 |
| PAK1/2/3 (Phospho-Ser141) | 0.99 | 1.19 | 1.17 |
| PAK1/2/3 (Phospho-Thr423/402/421) | 0.55 | 2.91^▲▲^ | 1.59 |
| PAK2 (Phospho-Ser192) | 1.62 | 0.59 | 0.95 |
| PAK3 (Phospho-Ser154) | 0.79 | 1.54 | 1.22 |
| Paxillin (Phospho-Tyr118) | 0.89 | 1.09 | 0.97 |
| Paxillin (Phospho-Tyr31) | 0.98 | 1.73 | 1.70 |
| PDGFR alpha (Phospho-Tyr849) | 0.95 | 0.95 | 0.90 |
| PDGFR beta (Phospho-Tyr1021) | 0.99 | 0.76 | 0.75 |
| PDGFR beta (Phospho-Tyr740) | 0.56 | 1.46^▲^ | 0.82 |
| PDGFR beta (Phospho-Tyr751) | 0.62 | 1.96^▲^ | 1.21 |
| PDK1 (Phospho-Ser241) | 0.84 | 1.36 | 1.13 |
| PEA-15 (Phospho-Ser116) | 0.80* | 1.09 | 0.88 |
| PECAM-1 (Phospho-Tyr713) | 0.68 | 2.55^▲▲^ | 1.73 |
| PI3-kinase p85-subunit alpha/gamma (Phospho-Tyr467/Tyr199) | 1.08 | 0.93 | 1.00 |
| Pim-1 (Phospho-Tyr309) | 1.02 | 0.99 | 1.01 |
| PKA CAT (Phospho-Thr197) | 1.06 | 0.84 | 0.89 |
| PKC alpha (Phospho-Tyr657) | 0.84 | 1.13 | 0.95 |
| PKC alpha/beta II (Phospho-Thr638) | 1.05 | 0.98 | 1.03 |
| PKC beta/PKCB (Phospho-Ser661) | 1.24 | 0.86 | 1.07 |
| PKC delta (Phospho-Ser645) | 0.79 | 2.35 | 1.85* |
| PKC delta (Phospho-Thr505) | 0.98 | 1.07 | 1.06 |
| PKC epsilon (Phospho-Ser729) | 0.63 | 1.60^▲^ | 1.00 |
| PKC pan activation site (Phospho) | 0.98 | 0.91 | 0.89 |
| PKC theta (Phospho-Ser676) | 0.72 | 1.19 | 0.86 |
| PKC zeta (Phospho-Thr410) | 1.34 | 0.83 | 1.11 |
| PKC zeta (Phospho-Thr560) | 0.83 | 1.30 | 1.08 |
| PKD1/PKC mu (Phospho-Ser205) | 1.01 | 1.12 | 1.13 |
| PKD1/PKC mu (Phospho-Ser910) | 0.81* | 1.21 | 0.98 |
| PKD1/PKC mu (Phospho-Tyr463) | 0.65 | 2.03^▲^ | 1.31* |
| PKD2 (Phospho-Ser876) | 1.42 | 0.78 | 1.11 |
| PKR (Phospho-Thr446) | 1.08 | 1.33 | 1.43 |
| PKR (Phospho-Thr451) | 0.45* | 2.25^▲^ | 1.02 |
| PLC beta3 (Phospho-Ser1105) | 0.64 | 1.52 | 0.97 |
| PLC beta3 (Phospho-Ser537) | 0.95 | 0.98 | 0.93 |
| PLCG1 (Phospho-Tyr771) | 1.19 | 0.70 | 0.84 |
| PLCG1 (Phospho-Tyr783) | 1.05 | 0.66 | 0.69 |
| PLCG2 (Phospho-Tyr1217) | 0.87 | 1.13 | 0.99 |
| PLCG2 (Phospho-Tyr753) | 0.83 | 1.32 | 1.10 |
| PLD1 (Phospho-Ser561) | 0.90 | 1.35^▲^ | 1.21* |
| PLK1 (Phospho-Thr210) | 0.29* | 3.96^▲^ | 1.16* |
| PP1 alpha (Phospho-Thr320) | 1.00 | 1.20 | 1.20* |
| PP2A-alpha (Phospho-Tyr307) | 1.15 | 1.14 | 1.31* |
| PPAR-BP (Phospho-Thr1457) | 0.74 | 1.46 | 1.08 |
| PPAR-gamma (Phospho-Ser112) | 0.31 | 6.37^▲^ | 1.99* |
| Progesterone Receptor (Phospho-Ser190) | 0.64 | 1.77^▲^ | 1.14 |
| PTEN (Phospho-Ser370) | 0.94 | 1.18 | 1.12 |
| PTEN (Phospho-Ser380) | 0.86 | 1.50^▲^ | 1.29 |
| PTEN (Phospho-Ser380/Thr382/Thr383) | 0.81 | 1.39 | 1.13 |
| Pyk2 (Phospho-Tyr402) | 1.30 | 0.74 | 0.96 |
| Pyk2 (Phospho-Tyr580) | 1.11 | 1.50 | 1.66 |
| Pyk2 (Phospho-Tyr881) | 0.43 | 2.83 | 1.21 |
| Rac1/cdc42 (Phospho-Ser71) | 0.77 | 1.41^▲^ | 1.08 |
| Raf1 (Phospho-Ser259) | 0.62* | 1.68^▲^ | 1.04 |
| Raf1 (Phospho-Ser289) | 1.09* | 0.90 | 0.99 |
| Raf1 (Phospho-Ser296) | 1.33 | 0.98 | 1.31 |
| Raf1 (Phospho-Ser338) | 0.80 | 1.40 | 1.12* |
| Raf1 (Phospho-Ser43) | 0.78* | 1.40^▲^ | 1.10 |
| Raf1 (Phospho-Ser621) | 1.03 | 1.18 | 1.23 |
| Raf1 (Phospho-Tyr341) | 1.01 | 0.95 | 0.96 |
| Ras-GRF1 (Phospho-Ser916) | 0.89 | 1.16 | 1.03 |
| Rb (Phospho-Ser608) | 0.92 | 0.99 | 0.91 |
| Rb (Phospho-Ser780) | 0.66 | 1.53 | 1.01 |
| Rb (Phospho-Ser795) | 1.12 | 1.24 | 1.40 |
| Rb (Phospho-Ser807) | 0.76 | 1.67^▲^ | 1.27 |
| Rb (Phospho-Ser811) | 0.56 | 2.31^▲^ | 1.29 |
| Rel (Phospho-Ser503) | 1.15 | 1.19 | 1.37 |
| RelB (Phospho-Ser552) | 0.83 | 0.84^▲^ | 0.70 |
| Ret (Phospho-Tyr905) | 1.56 | 0.93 | 1.45 |
| Rho/Rac guanine nucleotide exchange factor 2 (Phospho-Ser885) | 1.07 | 1.17 | 1.25 |
| RSK1/2/3/4 (Phospho-Ser221/227/218/232) | 1.04 | 1.27 | 1.32* |
| RyR2 (Phospho-Ser2808) | 1.31 | 0.88 | 1.15 |
| S6 Ribosomal Protein (Phospho-Ser235) | 0.75 | 1.34^▲^ | 1.00 |
| SAPK/JNK (Phospho-Thr183) | 1.19 | 0.85 | 1.01 |
| SAPK/JNK (Phospho-Tyr185) | 0.53** | 1.80^▲▲^ | 0.95 |
| Shc (Phospho-Tyr349) | 0.40 | 2.82 | 1.14 |
| Shc (Phospho-Tyr427) | 0.56 | 1.92^▲^ | 1.08 |
| SHP-2 (Phospho-Tyr542) | 1.17 | 0.94 | 1.10 |
| SHP-2 (Phospho-Tyr580) | 1.12 | 0.95 | 1.06 |
| SLP-76 (Phospho-Tyr128) | 0.74 | 1.67 | 1.23 |
| Smad1 (Phospho-Ser187) | 0.62 | 2.07 | 1.28 |
| Smad1 (Phospho-Ser465) | 0.87 | 1.92 | 1.67 |
| Smad2 (Phospho-Ser250) | 1.17 | 0.75^▲^ | 0.88 |
| Smad2 (Phospho-Ser467) | 0.33 | 3.33^▲▲^ | 1.09 |
| Smad2 (Phospho-Thr220) | 0.90 | 1.38 | 1.25 |
| Smad2/3 (Phospho-Thr8) | 1.21 | 0.79 | 0.95 |
| Smad3 (Phospho-Ser204) | 0.61* | 1.78^▲^ | 1.09 |
| Smad3 (Phospho-Ser213) | 1.61 | 0.43 | 0.69 |
| Smad3 (Phospho-Ser425) | 1.07 | 0.96 | 1.03 |
| Smad3 (Phospho-Thr179) | 0.73** | 1.43 | 1.04 |
| SMC1 (Phospho-Ser957) | 0.83 | 1.09 | 0.91 |
| SP1 (Phospho-Thr739) | 0.71 | 1.34 | 0.94 |
| Src (Phospho-Ser75) | 0.35 | 3.87 | 1.36 |
| Src (Phospho-Tyr418) | 0.44 | 3.68 | 1.61 |
| Src (Phospho-Tyr529) | 0.73** | 1.46^▲▲^ | 1.07** |
| SREBP-1 (Phospho-Ser439) | 0.51* | 1.77^▲^ | 0.90 |
| SRF (Phospho-Ser77) | 1.90 | 0.57 | 1.09 |
| SRF (Phospho-Ser99) | 0.64* | 2.27 | 1.45 |
| STAM2 (Phospho-Tyr192) | 1.06 | 0.70 | 0.75 |
| STAT1 (Phospho-Ser727) | 0.74 | 1.35 | 1.00 |
| STAT1 (Phospho-Tyr701) | 1.65** | 0.50^▲▲▲^ | 0.83** |
| STAT2 (Phospho-Tyr690) | 1.41* | 0.87 | 1.23 |
| STAT3 (Phospho-Ser727) | 1.34* | 0.91 | 1.22 |
| STAT3 (Phospho-Tyr705) | 1.21 | 0.88 | 1.07 |
| STAT4 (Phospho-Tyr693) | 1.07 | 0.94 | 1.00 |
| STAT5A (Phospho-Ser780) | 0.80 | 1.62^▲^ | 1.29 |
| STAT5A (Phospho-Tyr694) | 1.29 | 0.67 | 0.86 |
| STAT5B (Phospho-Ser731) | 0.57 | 1.43 | 0.82 |
| STAT6 (Phospho-Thr645) | 0.94 | 1.80^▲▲^ | 1.69* |
| STAT6 (Phospho-Tyr641) | 0.84 | 1.09 | 0.91 |
| Stathmin 1 (Phospho-Ser15) | 0.76 | 1.22 | 0.93 |
| Stathmin 1 (Phospho-Ser24) | 1.58* | 1.00 | 1.58* |
| Stathmin 1 (Phospho-Ser37) | 1.17 | 1.45 | 1.70 |
| Survivin (Phospho-Thr117) | 1.02 | 1.36 | 1.38* |
| SYK (Phospho-Tyr348) | 0.66* | 1.32^▲^ | 0.88* |
| SYK (Phospho-Tyr525) | 0.46 | 3.96^▲^ | 1.80** |
| Synapsin (Phospho-Ser62) | 1.18 | 1.01 | 1.19 |
| Synapsin (Phospho-Ser9) | 1.20 | 0.94 | 1.13 |
| Synaptotagmin (Phospho-Ser309) | 0.49* | 1.95^▲^ | 0.96 |
| Synaptotagmin (Phospho-Thr202) | 0.65 | 1.46^▲▲^ | 0.95 |
| Synuclein alpha (Phospho-Tyr125) | 1.00 | 0.97 | 0.97 |
| Synuclein alpha (Phospho-Tyr133) | 0.84 | 1.43 | 1.20 |
| Tau (Phospho-Ser214) | 0.70 | 1.73^▲^ | 1.20 |
| Tau (Phospho-Ser235) | 2.27 | 0.64 | 1.45 |
| Tau (Phospho-Ser262) | 0.99 | 1.35^▲▲^ | 1.33* |
| Tau (Phospho-Ser356) | 0.74 | 2.38^▲^ | 1.76* |
| Tau (Phospho-Ser396) | 0.46* | 3.18^▲^ | 1.47 |
| Tau (Phospho-Ser404) | 0.91 | 1.17^▲^ | 1.06 |
| Tau (Phospho-Ser422) | 2.42 | 0.12^▲^ | 0.30 |
| Tau (Phospho-Thr181) | 1.37* | 0.73^▲^ | 1.00 |
| Tau (Phospho-Thr205) | 0.85 | 1.35^▲^ | 1.16 |
| Tau (Phospho-Thr212) | 1.10 | 1.76^▲^ | 1.93* |
| Tau (Phospho-Thr231) | 1.42* | 0.65^▲^ | 0.92 |
| TIF-IA (Phospho-Ser649) | 1.32 | 0.62^▲▲^ | 0.82 |
| TOP2A/DNA topoisomerase II (Phospho-Ser1106) | 0.73 | 1.86 | 1.36 |
| Trk B (Phospho-Tyr515) | 0.79 | 1.58^▲^ | 1.24 |
| Tuberin/TSC2 (Phospho-Ser939) | 0.58 | 2.15 | 1.25 |
| Tuberin/TSC2 (Phospho-Thr1462) | 1.32 | 0.75 | 0.99 |
| TYK2 (Phospho-Tyr1054) | 1.19 | 1.02 | 1.20* |
| Tyrosine Hydroxylase (Phospho-Ser19) | 0.68* | 1.74^▲^ | 1.19 |
| Tyrosine Hydroxylase (Phospho-Ser31) | 0.64 | 1.74^▲^ | 1.11 |
| Tyrosine Hydroxylase (Phospho-Ser40) | 0.47** | 2.37^▲^ | 1.12 |
| Tyrosine Hydroxylase (Phospho-Ser8) | 0.95 | 2.00^▲^ | 1.91 |
| VASP (Phospho-Ser157) | 1.10 | 0.89 | 0.98 |
| VASP (Phospho-Ser238) | 0.88 | 1.20 | 1.06 |
| VAV1 (Phospho-Tyr174) | 1.09 | 0.96 | 1.05 |
| VAV2 (Phospho-Tyr142) | 1.23 | 0.70 | 0.86* |
| VEGFR1 (Phospho-Tyr1333) | 0.71* | 1.65 | 1.17 |
| VEGFR2 (Phospho-Tyr1054) | 0.93 | 0.86 | 0.80 |
| VEGFR2 (Phospho-Tyr1059) | 1.03 | 1.02 | 1.05 |
| VEGFR2 (Phospho-Tyr1175) | 0.53 | 1.47^▲^ | 0.77 |
| VEGFR2 (Phospho-Tyr1214) | 0.86 | 1.80 | 1.55** |
| VEGFR2 (Phospho-Tyr951) | 0.58 | 4.78 | 2.78 |
| Vinculin (Phospho-Tyr821) | 1.92 | 0.55 | 1.05 |
| WASP (Phospho-Tyr290) | 0.75 | 1.32 | 1.00 |
| WAVE1 (Phospho-Tyr125) | 0.98 | 1.07 | 1.05 |
| WEE1 (Phospho-Ser53) | 1.00 | 1.14^▲^ | 1.14* |
| XIAP (Phospho-Ser87) | 0.79 | 1.51 | 1.20 |
| Zap-70 (Phospho-Tyr292) | 0.86 | 1.51 | 1.30* |
| Zap-70 (Phospho-Tyr319) | 1.05 | 1.21^▲^ | 1.27 |
| Zap-70 (Phospho-Tyr493) | 0.41* | 2.75^▲^ | 1.11 |

**Supplementary Table 1.** The phosphorylation changes of overall proteins after BCP modeling or EA treatment. The three-line table showed the *P* value significance of difference comparisons between groups. The overall 584 phosphorylated proteins were included. BCP/Con: the fold change of protein phosphorylation in the BCP group compared to the control group; BCP+EA/BCP: the fold change of protein phosphorylation in the BCP+EA group compared to the BCP group; BCP+EA/Con: the fold change of protein phosphorylation in the BCP+EA group compared to the control group. * *P* < 0.05, ** *P* < 0.01, *** *P* < 0.001, compared with the Control group; ▲ *P* < 0.05, ▲▲ *P* < 0.01, ▲▲▲*P* < 0.001, compared with the BCP group. Con: Control.

**Supplementary Table 2.**

| Protein List | BCP/Con | BCP+EA/BCP | BCP+EA/Con |
| --- | --- | --- | --- |
| 4E-BP1 (Phospho-Ser65) | 0.39 | 4.93^▲▲^ | 1.92 |
| Abl1 (Phospho-Thr754/735) | 2.36 | 0.36 | 0.86 |
| AFX/FOXO4 (Phospho-Ser197) | 0.59* | 2.27^▲^ | 1.33 |
| AKT1 (Phospho-Tyr326) | 0.39 | 4.17^▲^ | 1.64 |
| Androgen Receptor (Phospho-Ser650) | 0.58* | 1.94^▲^ | 1.13* |
| ATRIP (Phospho-Ser68/72) | 0.51 | 3.27^▲^ | 1.67 |
| BAD (Phospho-Ser155) | 0.62* | 2.15^▲▲^ | 1.33* |
| BCL-XL (Phospho-Thr47) | 0.56 | 3.21^▲^ | 1.80 |
| CaMK2A (Phospho-Thr286) | 0.51* | 2.06^▲^ | 1.04 |
| Caspase 3 (Phospho-Ser150) | 0.50 | 1.78 | 0.89 |
| Caspase 9 (Phospho-Thr125) | 0.34** | 2.19^▲^ | 0.75* |
| Catenin beta (Phospho-Ser37) | 0.48 | 2.23 | 1.06 |
| Catenin beta (Phospho-Tyr654) | 0.51* | 1.79^▲^ | 0.91 |
| CD5 (Phospho-Tyr453) | 0.50* | 2.48^▲^ | 1.23** |
| CDC25A (Phospho-Ser124) | 1.60 | 0.52 | 0.83 |
| CDC25A (Phospho-Ser75) | 1.62 | 0.48 | 0.78 |
| CDC25B (Phospho-Ser353) | 1.82* | 0.50^▲^ | 0.91 |
| CDC25C (Phospho-Ser216) | 0.31*** | 4.49^▲▲^ | 1.40* |
| CDK1/CDC2 (Phospho-Thr14) | 2.55*** | 0.44^▲^ | 1.13 |
| c-Jun (Phospho-Thr93) | 1.63 | 0.60 | 0.99 |
| CREB (Phospho-Ser133) | 2.13** | 0.59^▲▲^ | 1.25** |
| Cyclin E1 (Phospho-Thr77) | 0.52** | 4.09 | 2.13 |
| DAB1 (Phospho-Tyr232) | 0.58* | 1.99^▲^ | 1.14 |
| DNA-PK (Phospho-Thr2647) | 2.13 | 0.35 | 0.74* |
| Dok-1 (Phospho-Tyr398) | 0.55* | 1.92^▲^ | 1.06 |
| DYN1 (Phospho-Ser774) | 0.51 | 3.21 | 1.63 |
| E2F1 (Phospho-Thr433) | 0.54* | 2.17 | 1.17 |
| EGFR (Phospho-Ser1070) | 0.53* | 2.50^▲^ | 1.33 |
| Elk1 (Phospho-Ser389) | 0.41* | 2.74^▲^ | 1.12* |
| Ephrin B2 (Phospho-Tyr330) | 0.54* | 2.35^▲^ | 1.26 |
| Estrogen Receptor-alpha (Phospho-Ser104) | 0.58 | 2.01 | 1.17 |
| ETK (Phospho-Tyr40) | 0.61 | 1.74 | 1.06 |
| Ezrin (Phospho-Thr566) | 0.36* | 3.88^▲^ | 1.40* |
| FAK (Phospho-Tyr397) | 0.46 | 2.29 | 1.05 |
| FER (Phospho-Tyr402) | 0.59 | 1.92 | 1.12 |
| GluR1 (Phospho-Ser849) | 0.42* | 7.28^▲^ | 3.05 |
| HDAC2 (Phospho-Ser394) | 1.62** | 0.56^▲^ | 0.92 |
| HDAC6 (Phospho-Ser22) | 0.46* | 2.65^▲^ | 1.22 |
| HER4/ErbB4 (Phospho-Tyr1284) | 0.62 | 1.85 | 1.14 |
| Histone H2A.X (Phospho-Ser139) | 0.60 | 1.74 | 1.04 |
| HSP90B (Phospho-Ser254) | 0.55 | 1.85^▲^ | 1.02 |
| IGF1R (Phospho-Tyr1161) | 1.83** | 0.60 | 1.10 |
| IKK-beta (Phospho-Tyr199) | 0.51 | 2.57^▲▲▲^ | 1.30 |
| IRS-1 (Phospho-Ser307) | 0.54** | 1.77^▲^ | 0.96 |
| IRS-1 (Phospho-Ser323) | 0.51 | 2.38^▲▲^ | 1.22 |
| IRS-1 (Phospho-Ser639) | 0.32* | 3.29^▲^ | 1.07 |
| JAK2 (Phospho-Tyr1007) | 0.62 | 2.10 | 1.31 |
| JAK2 (Phospho-Tyr221) | 0.60* | 1.89^▲^ | 1.13 |
| Lamin A/C (Phospho-Ser392) | 0.39* | 4.79^▲▲^ | 1.85** |
| LAT (Phospho-Tyr171) | 0.59 | 1.84 | 1.08 |
| LAT (Phospho-Tyr191) | 0.60* | 1.72^▲^ | 1.03 |
| LYN (Phospho-Tyr507) | 0.17 | 6.06 | 1.00 |
| MAP3K8/COT (Phospho-Thr290) | 0.59* | 2.06^▲▲^ | 1.22* |
| MEF2A (Phospho-Ser408) | 0.62* | 1.93^▲^ | 1.20 |
| MEK1 (Phospho-Ser298) | 0.25* | 4.02 | 1.01 |
| Merlin (Phospho-Ser10) | 0.43 | 6.15^▲^ | 2.64* |
| Met (Phospho-Tyr1003) | 0.49 | 2.11 | 1.03 |
| MKK3/MAP2K3 (Phospho-Ser189) | 0.58* | 1.72^▲^ | 1.00 |
| MKK7/MAP2K7 (Phospho-Ser271) | 0.58 | 1.93^▲^ | 1.12 |
| Mnk1 (Phospho-Thr385) | 0.46* | 1.92^▲^ | 0.88 |
| mTOR (Phospho-Ser2481) | 0.41* | 2.31^▲^ | 0.96 |
| NFkB-p105/p50 (Phospho-Ser927) | 0.59 | 3.55 | 2.08 |
| NFkB-p65 (Phospho-Ser311) | 0.45* | 2.44^▲▲^ | 1.09 |
| NFkB-p65 (Phospho-Ser468) | 1.86 | 0.48 | 0.90 |
| NMDAR2B (Phospho-Tyr1472) | 0.58 | 2.83^▲^ | 1.64 |
| Opioid Receptor (Phospho-Ser375) | 1.65 | 0.53^▲^ | 0.87* |
| p38 MAPK (Phospho-Tyr182) | 0.54 | 3.20^▲▲^ | 1.72 |
| p53 (Phospho-Ser33) | 0.60 | 1.98 | 1.18 |
| P70S6K (Phospho-Ser411) | 0.60* | 2.01^▲^ | 1.20 |
| PAK1/2 (Phospho-Ser199) | 0.56 | 1.91 | 1.08 |
| PAK1/2/3 (Phospho-Thr423/402/421) | 0.55 | 2.91^▲▲^ | 1.59 |
| PAK2 (Phospho-Ser192) | 1.62 | 0.59 | 0.95 |
| PDGFR beta (Phospho-Tyr751) | 0.62 | 1.96^▲^ | 1.21 |
| PKR (Phospho-Thr451) | 0.45* | 2.25^▲^ | 1.02 |
| PLK1 (Phospho-Thr210) | 0.29* | 3.96^▲^ | 1.16* |
| PPAR-gamma (Phospho-Ser112) | 0.31 | 6.37^▲^ | 1.99* |
| Pyk2 (Phospho-Tyr881) | 0.43 | 2.83 | 1.21 |
| Raf1 (Phospho-Ser259) | 0.62* | 1.68^▲^ | 1.04 |
| Rb (Phospho-Ser811) | 0.56 | 2.31^▲^ | 1.29 |
| SAPK/JNK (Phospho-Tyr185) | 0.53** | 1.80^▲▲^ | 0.95 |
| Shc (Phospho-Tyr349) | 0.40 | 2.82 | 1.14 |
| Shc (Phospho-Tyr427) | 0.56 | 1.92^▲^ | 1.08 |
| Smad1 (Phospho-Ser187) | 0.62 | 2.07 | 1.28 |
| Smad2 (Phospho-Ser467) | 0.33 | 3.33^▲▲^ | 1.09 |
| Smad3 (Phospho-Ser204) | 0.61* | 1.78^▲^ | 1.09 |
| Smad3 (Phospho-Ser213) | 1.61 | 0.43 | 0.69 |
| Src (Phospho-Ser75) | 0.35 | 3.87 | 1.36 |
| Src (Phospho-Tyr418) | 0.44 | 3.68 | 1.61 |
| SREBP-1 (Phospho-Ser439) | 0.51* | 1.77^▲^ | 0.90 |
| SRF (Phospho-Ser77) | 1.90 | 0.57 | 1.09 |
| STAT1 (Phospho-Tyr701) | 1.65** | 0.50^▲▲▲^ | 0.83** |
| SYK (Phospho-Tyr525) | 0.46 | 3.96^▲^ | 1.80** |
| Synaptotagmin (Phospho-Ser309) | 0.49* | 1.95^▲^ | 0.96 |
| Tau (Phospho-Ser396) | 0.46* | 3.18^▲^ | 1.47 |
| Tau (Phospho-Ser422) | 2.42 | 0.12^▲^ | 0.30 |
| Tuberin/TSC2 (Phospho-Ser939) | 0.58 | 2.15 | 1.25 |
| Tyrosine Hydroxylase (Phospho-Ser40) | 0.47** | 2.37^▲^ | 1.12 |
| VEGFR2 (Phospho-Tyr951) | 0.58 | 4.78 | 2.78 |
| Vinculin (Phospho-Tyr821) | 1.92 | 0.55 | 1.05 |
| Zap-70 (Phospho-Tyr493) | 0.41* | 2.75^▲^ | 1.11 |

**Supplementary Table 2.** The phosphorylation changes of DEPs co-regulated by both BCP modeling and EA treatment. The three-line table showed the *P* value significance of difference comparisons between groups. The DEPs co-regulated by both BCP modeling and EA treatment were included. BCP/Con: the fold change of protein phosphorylation in the BCP group compared to the control group; BCP+EA/BCP: the fold change of protein phosphorylation in the BCP+EA group compared to the BCP group; BCP+EA/Con: the fold change of protein phosphorylation in the BCP+EA group compared to the control group. * *P* < 0.05, ** *P* < 0.01, *** *P* < 0.001, compared with the Control group; ▲ *P* < 0.05, ▲▲ *P* < 0.01, ▲▲▲*P* < 0.001, compared with the BCP group. Con: Control; DEPs: differential expressed phosphorylated proteins.

**Supplementary Figure 1.**

**
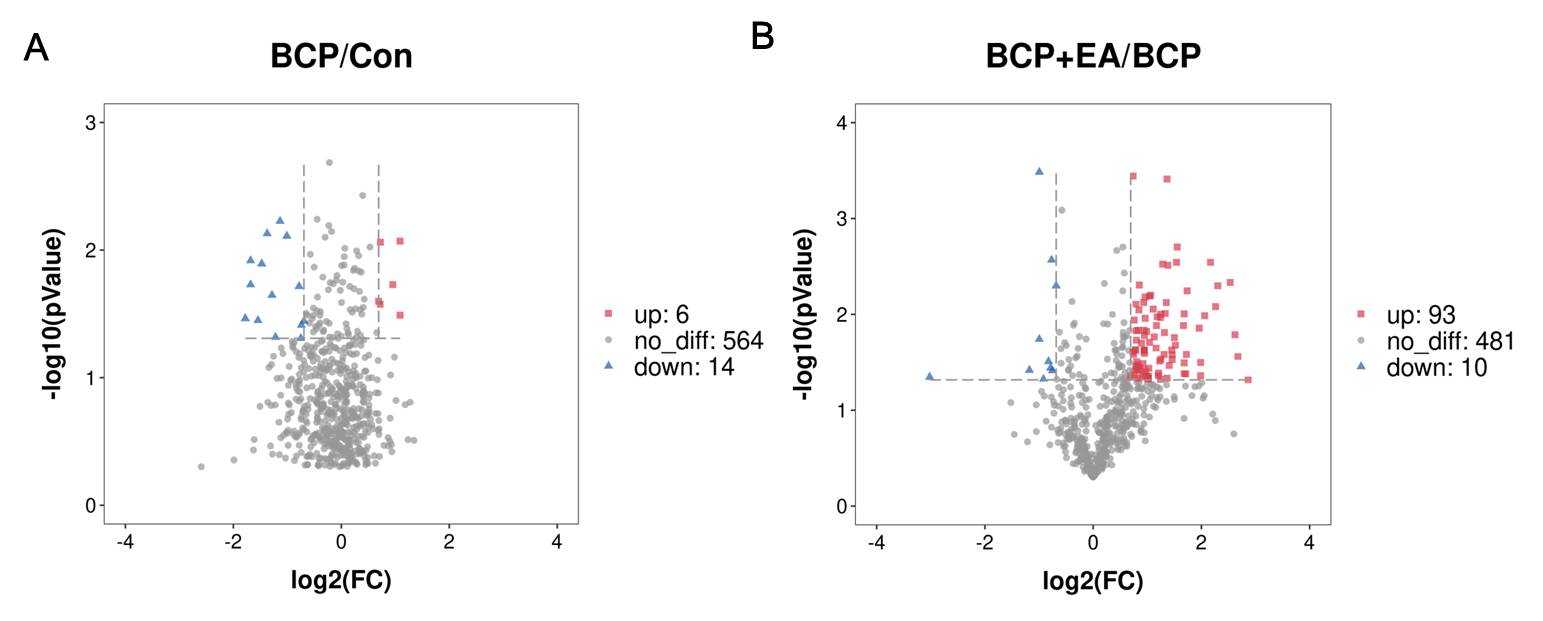
**

**Supplementary Figure 1.** The differential phosphorylation expressions of proteins after BCP modeling or EA treatment. **(A)** The volcano plot of protein phosphorylation changes after BCP modeling (BCP vs Con); **(B)** The volcano plot of protein phosphorylation changes after EA treatment (BCP+EA vs BCP). X‐axis: log2(fold change); Y‐axis: -log10(*P*‐value). The red squares represent phosphorylated proteins that were significantly up‐regulated; the blue triangles represent phosphorylated proteins that were significantly down‐regulated. Con: Control.

**Supplementary Figure 2.**


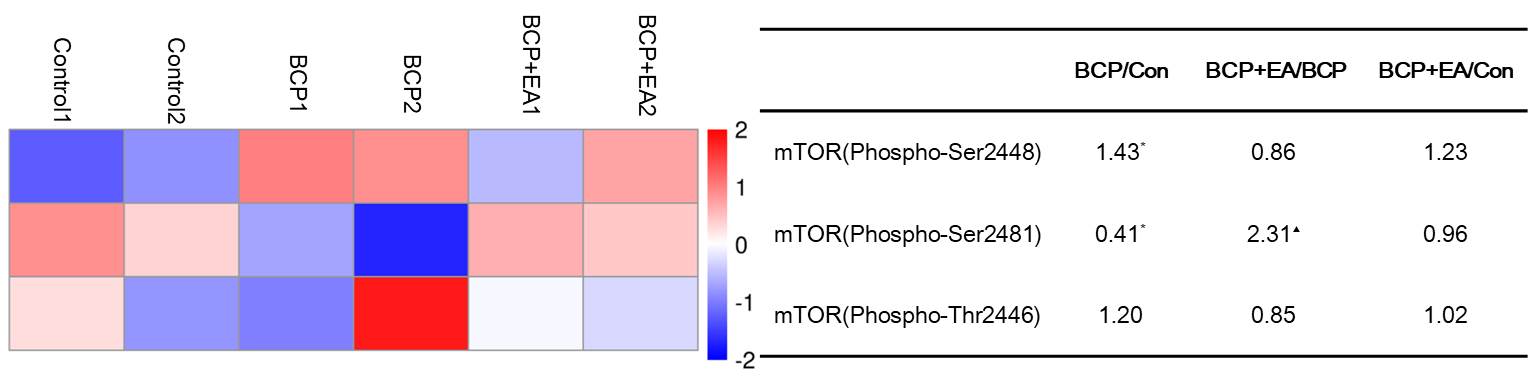


**Supplementary Figure 2.** The phosphoproteomic proﬁle of mTOR detected by Phosphor-explorer array PEX100 assay. Heat map was color-based on the phosphorylation level of mTOR at Thr2446, Ser2448 and Ser2481. The red color means higher expression while the blue color means lower expression. Control 1 and Control 2 represent the two different samples in the Control group and it is the same with others. The three-line table showed the p-value significance of difference comparisons between groups. BCP/Con: the fold change of protein phosphorylation in the BCP group compared to the control group; BCP+EA/BCP: the fold change of protein phosphorylation in the BCP+EA group compared to the BCP group; BCP+EA/Con: the fold change of protein phosphorylation in the BCP+EA group compared to the control group. * *P* < 0.05, compared with the Control group; ▲ *P* < 0.05, compared with the BCP group. Con: Control.

**References**

1.He D, Wu H, Xiang J, Ruan X, Peng P, Ruan Y, et al. Gut stem cell aging is driven by mTORC1 via a p38 MAPK-p53 pathway. *Nature Communications* (2020) 11(1). doi: 10.1038/s41467-019-13911-x. PubMed PMID: 31896747; PubMed Central PMCID: PMCPMC6940394.

2.Chen L, Kong R, Wu C, Wang S, Liu Z, Liu S, et al. Circ-MALAT1 Functions as Both an mRNA Translation Brake and a microRNA Sponge to Promote Self-Renewal of Hepatocellular Cancer Stem Cells. *Adv Sci (Weinh)* (2020) 7(4):1900949. doi: 10.1002/advs.201900949. PubMed PMID: 32099751; PubMed Central PMCID: PMCPMC7029649.
